# Supplementary material for: The structure of a Plasmodium vivax Tryptophan Rich Antigen domain suggests a lipid binding function for a pan-Plasmodium multi-gene family
Source: Nat Commun. 2023 Sep 14;14:5703. doi: 10.1038/s41467-023-40885-8 (PMC10502043; doi:10.1038/s41467-023-40885-8)
Supplement: Supplementary file 1 — Supplementary information [file 41467_2023_40885_MOESM1_ESM.pdf]

The structure of a *Plasmodium vivax* Tryptophan Rich Antigen domain suggests a lipid binding function for a pan-*Plasmodium* multi-gene family

Prasun Kundu[1#], Deboki Naskar[1#], Shannon J. McKie[1], Sheena Dass[2], Usheer Kanjee[2], Viola Introini[3], Marcelo U. Ferreira[4,5], Pietro Cicuta[3,] Manoj Duraisingh[2]*, Janet E. Deane[1]*, Julian C. Rayner[1]*

[1] Cambridge Institute for Medical Research, University of Cambridge, Cambridge, United Kingdom

[2] Department of Immunology and Infectious Diseases, Harvard T.H. Chan School of Public Health, Boston, Massachusetts, USA.

[3]Cavendish Laboratory, Department of Physics, University of Cambridge, Cambridge, United Kingdom

[4]Department of Parasitology, Institute of Biomedical Sciences, University of São Paulo, São Paulo, Brazil

[5]Global Health and Tropical Medicine, Associate Laboratory in Translation and Innovation Towards Global Health, LA-REAL, Institute of Hygiene and Tropical Medicine, NOVA University of Lisbon, Lisbon, Portugal

([#]contributed equally)

(*co-corresponding authors)
Julian C. Rayner: jcr1003@cam.ac.uk
Janet E. Deane: jed55@cam.ac.uk
Manoj Duraisingh: mduraisi@hsph.harvard.edu

Supplementary Figures and legends:

Supplementary Fig. 1

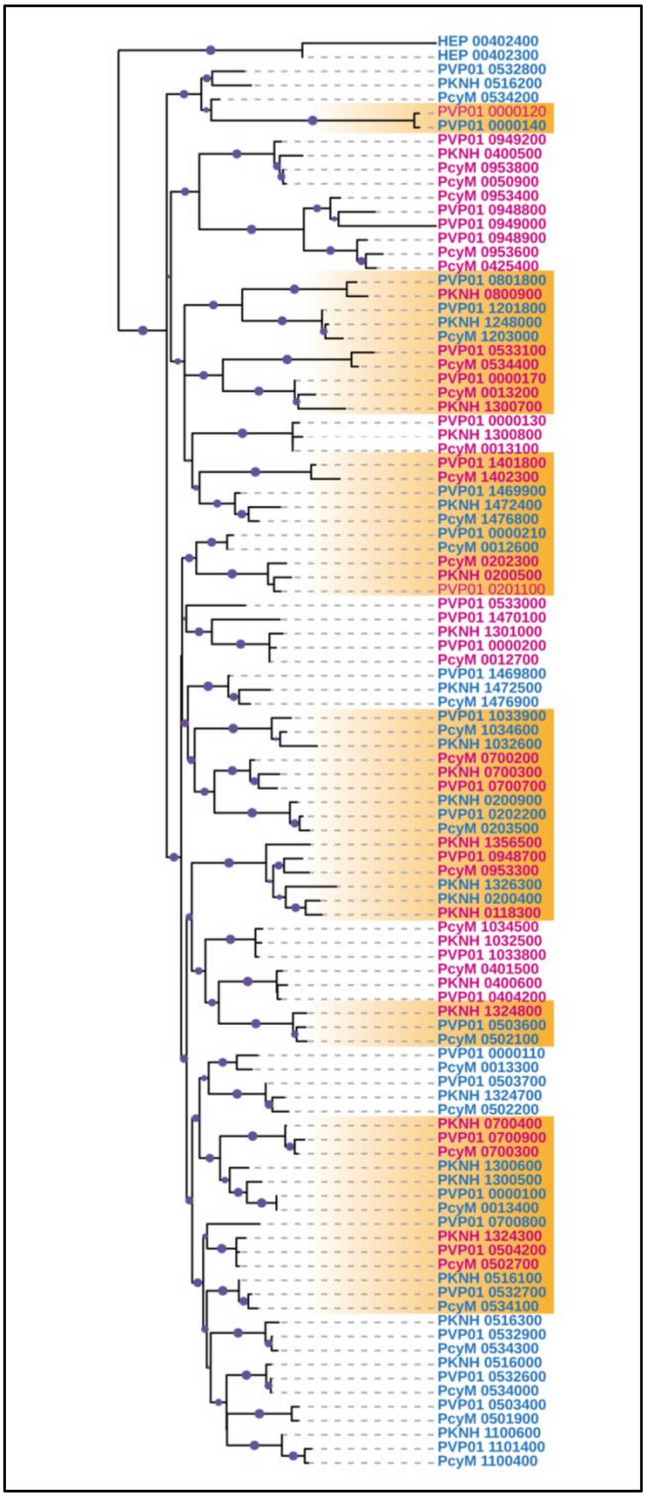

Supplementary Fig. 1: Phylogenetic tree of the TRAgs. Pv, Pk and Pc TRAgs (colour coded as Blue for -SP and magenta +SP) show their position on the phylogenetic tree. The phylogenetic tree was reconstructed using the maximum likelihood method with 100 bootstrap replicates. The scale for the bootstrap values is 0.25-1 represented with the size of the purple spherical symbol on each branch. The orange boxes highlight clusters where TRAgs with and without predicted SPs are grouped together. This suggests the SPs must have been gained or lost multiple times in the evolution of the family, which seems unlikely – issues with genome annotation, discussed in the text, seem more likely.

Supplementary Fig. 2

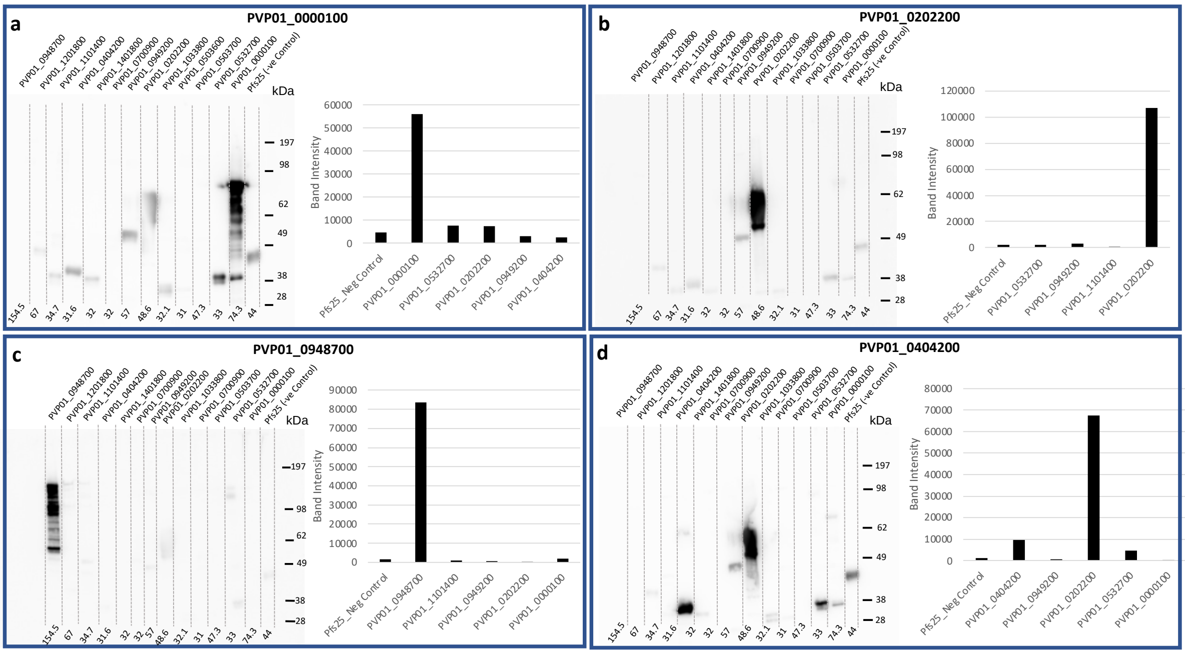

Supplementary Fig. 2: a, Cross reactivity of the IgGs raised against four PvTRAgs used in this study were assessed in a western blot. The PVP01_0000100 IgG were tested against a panel of other 13 different TRAgs including PVP01_0000100. Pfs25 used as a background control in the assay to quantify any reactivity against the tags. The band intensity for PVP01_0000100 were plotted to quantify the level of cross reactivity with other TRAgs. An overall very weak cross reactivity was evident for PVP01_0000100 IgGs with all other TRAgs. **b** & **c** Antibodies against PVP01_0202200 and PVP01_0948700 are found to be very weakly cross reactive as none of the other TRAgs have band intensities surpassed the background level. **d**, The antibodies against PVP01_0404200 are found to be weakly cross reactive towardsall other TRAgs except PVP01_0202200 which seems to be crossreacting strongly.

Supplementary Fig. 3

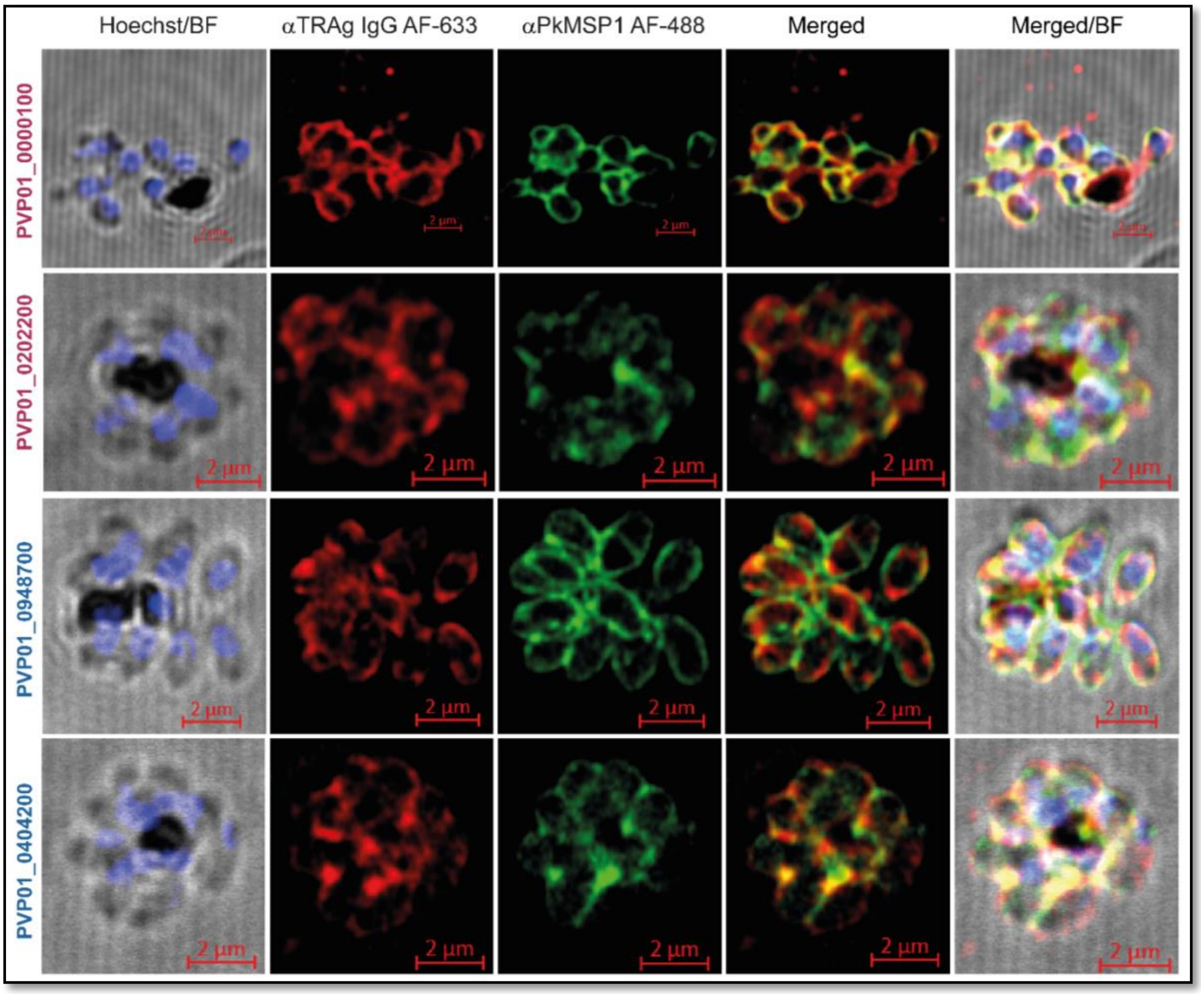

Supplementary Fig. 3: The Immunolocalization of the four TRAgs in *P. knowlesi* mature schizonts. The Rabbit antibodies raised against the 4 PvTRAgs (colour coded as magenta for –SP and Blue for +SP) are acting as primary antibody recognising their targets (Red) and its colocalised with PkMSP1 (Green) a surface marker for merozoites. Scale bar is 2uM. The data is a representation of n=3 independent experiment with similar observation.

Supplementary Fig. 4

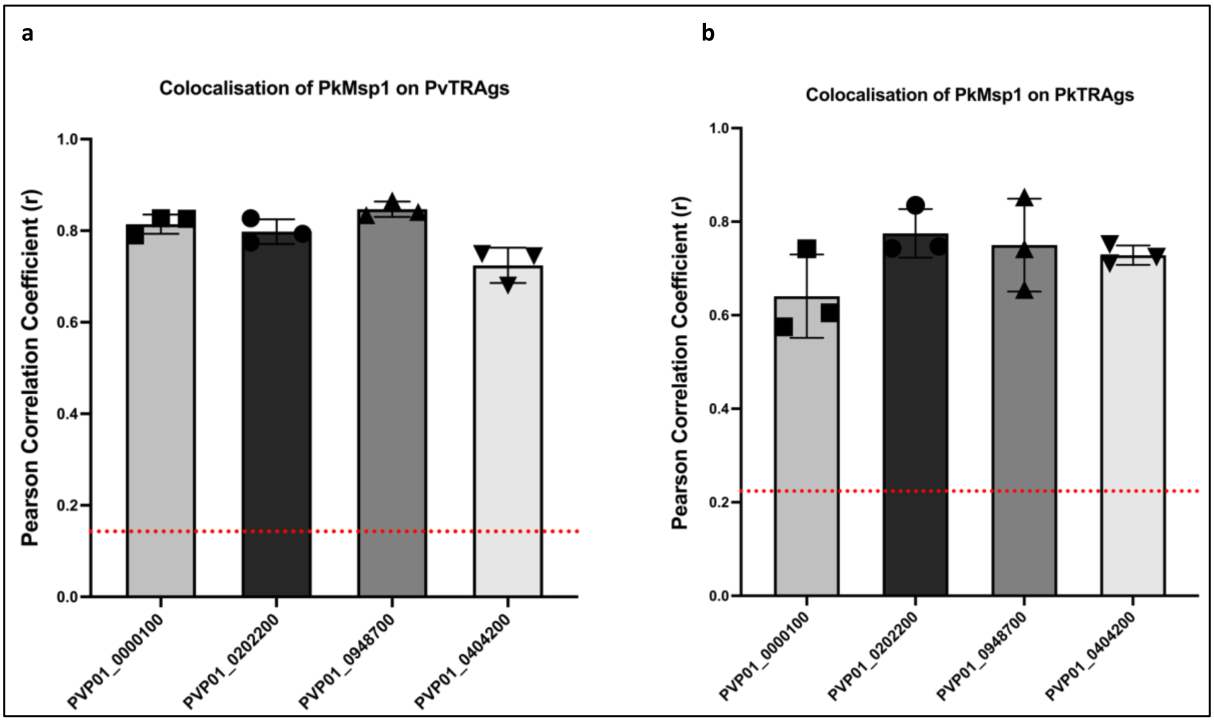

Supplementary Fig. 4: The colocalization of PkMSP1 with four PvTRAgs antibodies in *P. vivax* isolates (**a**) and in *P. knowlesi* wild type (**b**) was quantified by measuring the Pearson correlation coefficient, r. The background cut off (red dotted line) was determined by calculating the r value by superimposing the DNA stain (Hoechst) upon merozoite surface localised PvTRAgs (which are supposed not to be co-localised with each other). The r value were calculated from n=3 independent experiment and plotted as mean values (+/-SD).

Supplementary Fig. 5

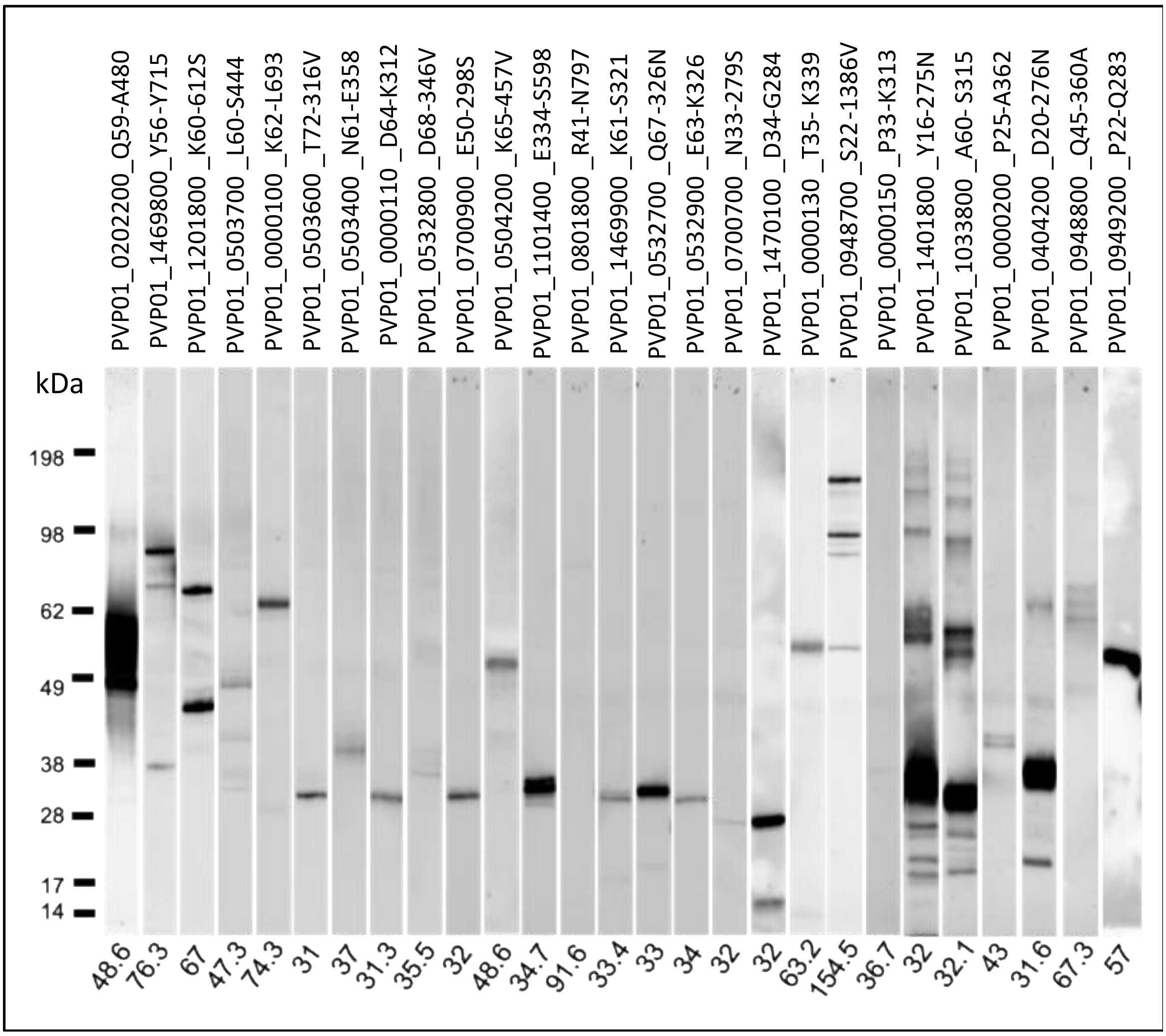

Supplementary Fig. 5: A western blot image of a panel of expressed recombinant PvTRAgs. Protein samples were collected from the HEK293E cell culture supernatant and resolved by 4-12% SDS-PAGE under reducing condition. The resolved proteins were blotted to a nitrocellulose membrane and probed using an anti-His-HRP antibody followed by developed using ECL substrate. The predicted molecular weights of the recombinant proteins are indicated below respective blot strips. The domain boundaries for all the ectodomains are mentioned along with the gene IDs on top of the blots. This blot is representative of n=2 independent experiment.

Supplementary Fig. 6

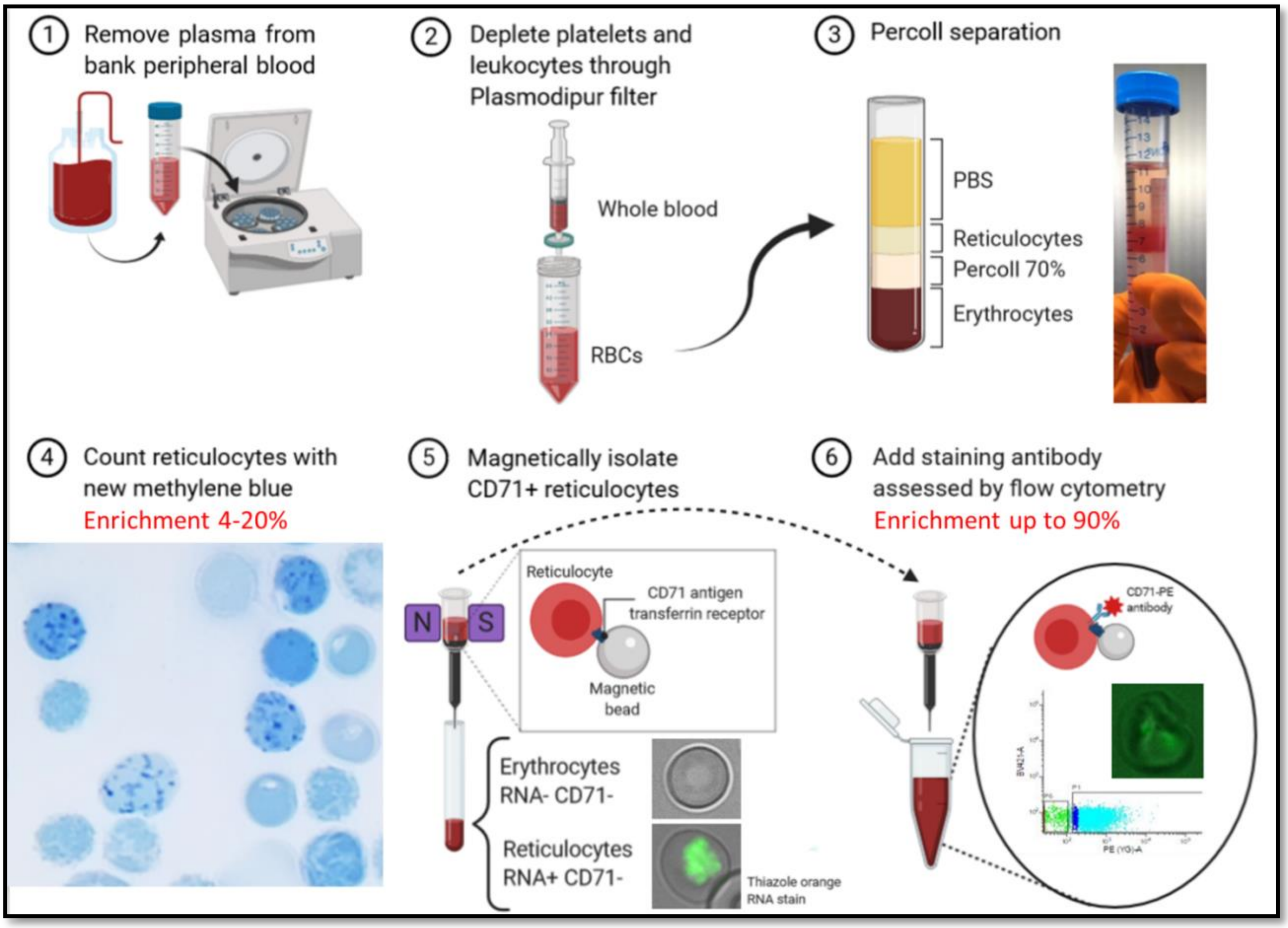

Supplementary Fig. 6: A step by step schematic diagram of the reticulocyte Isolation process starting from human whole blood. The figure is created with BioRender.com.

Supplementary Fig. 7

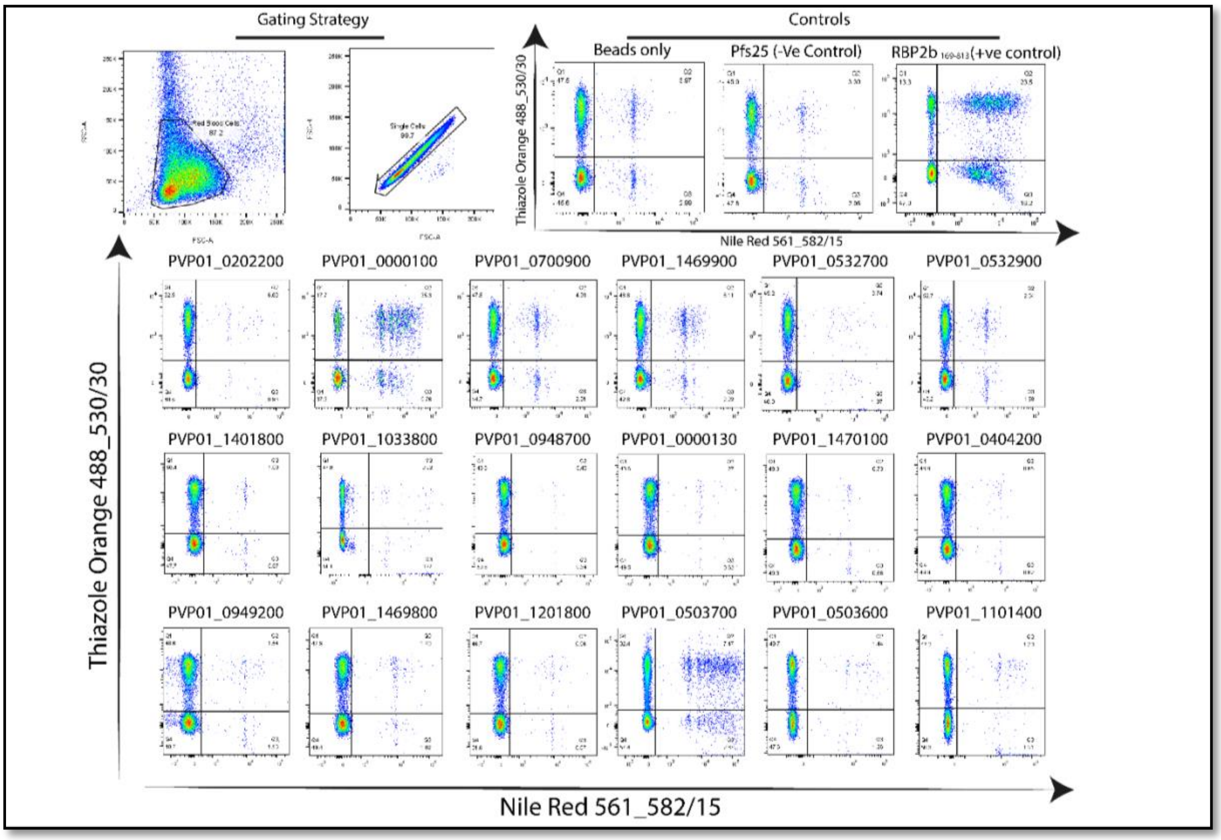

Supplementary Fig. 7: The gating strategy shown for the red blood cell binding assay and single cells were gated out to be considered for the binding assay. RBP2b$_{169\text{-}813}$ which has reported to have binding towards Cd71+ reticulocytes were used as positive control whereas Pfs25 a sexual stage specific antigen used as negative control for this binding assay. In the Y axis the population of the cell were distributed based on the presence of genetic material as Thiazole orange binds to the RNA present in immature reticulocytes compare to erythrocytes (Q1-Reticulocyte and Q4-Erythrocyte). On X axis the Nile red coated streptavidin beads bind to biotinylated proteins and the cells bound to the bead conjugated proteins are shifted towards right (reticuclocyte, Q2 and erythrocytes, Q3) due to the fluorescence emission in the red channel. PVP01_0000100 and PVP01_0503700 has shown the highest binding over 62% and 20% compared to the total retics population (Q1+Q2) and has shown to have a clear preference for retics over erythrocytes.

Supplementary Fig. 8

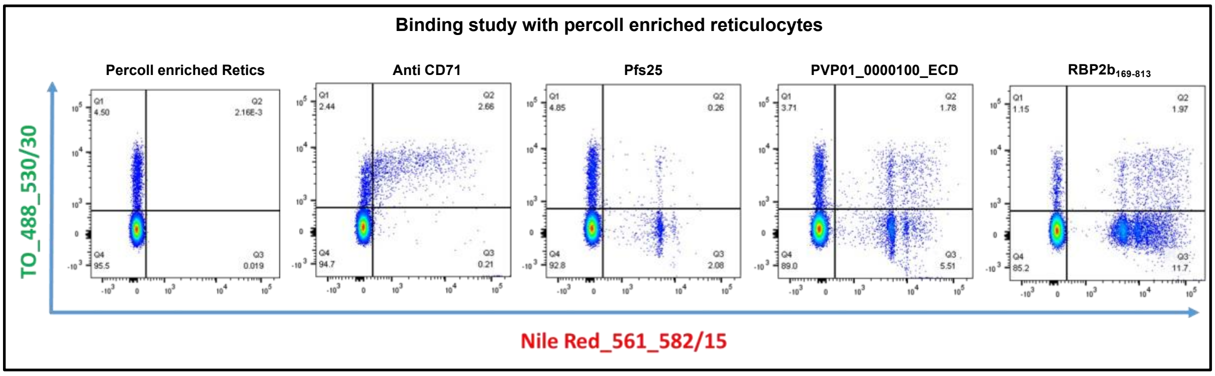

Supplementary Fig. 8: The red blood cell binding assay performed with percoll enriched reticulocytes. The reticulocyte's rRNAs get stained with Thiazole Orange (TO) whereas the erythrocytes remain unstained. The respective population (reticulocyte or erythrocyte) bound to protein coated streptavidin Nile red (NR) beads will shift to Q2 or Q3 quadrant (Q1: TO+/NR-,Q2: TO+/NR+, Q3: TO-/NR+ and Q4: TO-/NR-). N=2 independent experiment with n=2 technical replicate.

Supplementary Fig. 9

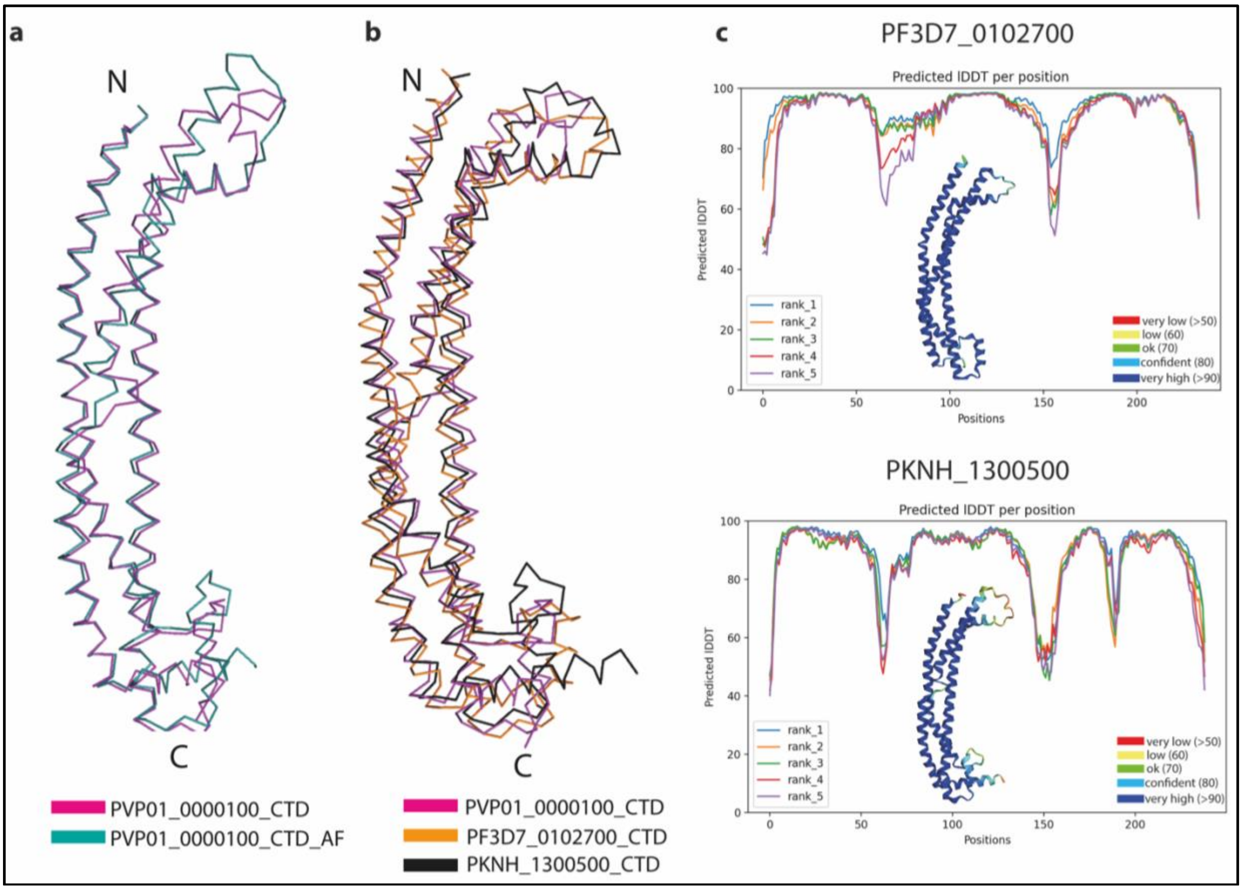

Supplementary Fig. 9: a Superposition of the experimentally determined structure of PVP01_0000100 CTD (magenta) with an Alphafold predicted model (green cyan) shows an overall high similarity (1.56 Å over 213 C-alpha atoms) supporting the use of predicted models of other TRAgs for comparison**. b,** Structural similarity of Pv/Pk/Pf orthologs**,** the superposition of the tryptophan rich domain of PVP01_0000100 with its orthologue in Pf (RMSD 2.87 Å over 179 C-alpha atoms) and Pk (RMSD 1.83 Å over 211 C-alpha atoms) suggests an inter species conservation of the C-terminal domain in the TRAg family. **c,** The pLDDT plots from AlphaFold showing a per-residue measure of local confidence score on a scale from 0 – 100. The core extended helical region is predicted with high confidence for all models. All five predicted models are nearly identical to each other, so the rank 1 model used here is representative of the predicted structures.

Supplementary Fig. 10

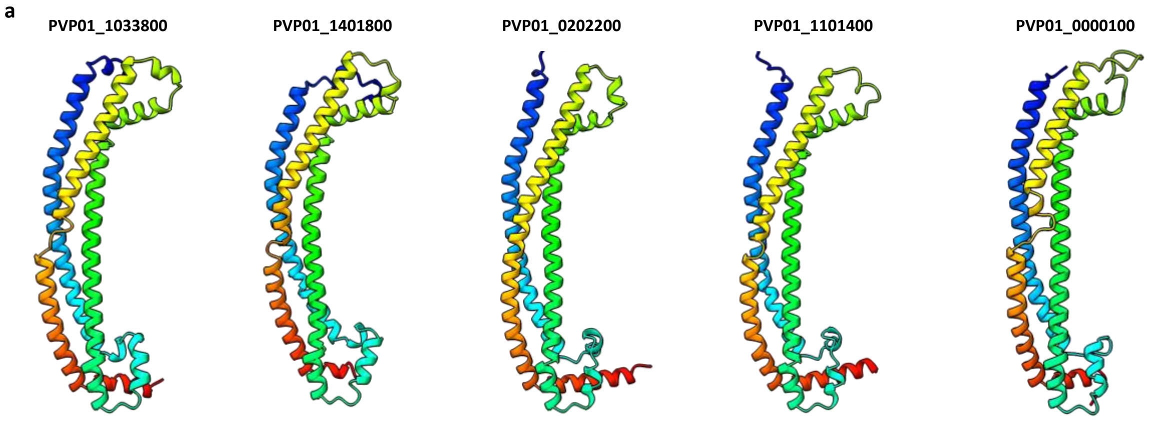

Gene ID	Residues aligned out of 232 residues in reference structure (PVP01_0000100)	RMSD (Å	Seq Identity (%)
PVP01_1033800	212	1.88	35
PVP01_1401800	203	2.13	28
PVP01_0202200	196	1.31	36.8
PVP01_1101400	195	1.48	42

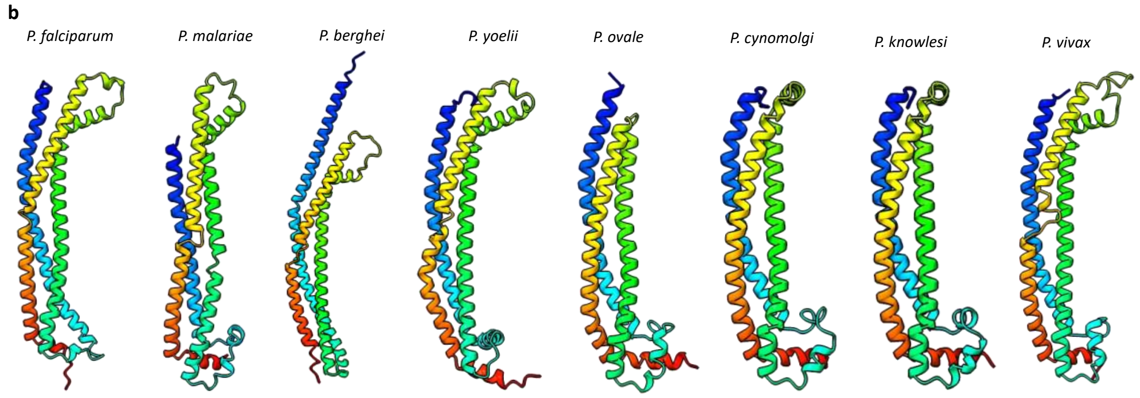

Gene ID	Residues aligned out of 232 residues in reference structure (PVP01_0000100)	RMSD (Å	Seq Identity (%)
PF3D7_1002200	181	1.93	26.5
PmUG01_00069200	185	4.00	21.7
PBANKA_0623300	178	2.16	28.6
PY17X_0701300	196	2.21	24.9
PocGF01_00099300	173	1.76	22.3
Pcym_0013100	160	2.66	25
PKNH_1300800	164	2.58	25.5

Supplementary Fig. 10: Structural conservation of the helical fold for TRAg domains in paralogues of *P. vivax* and across different Plasmodium species. a) Four different TRAgs were selected based on their position in the phylogenetic tree and with varied range of sequence identity. The C-terminal domains for each of these TRAgs, apart from PVP01_0000100, were modelled in Alphafold and superposed (Cα atoms) using Coot to generate the RMSD values listed in the table (*bottom*). **b)** Seven different TRAgs were selected from different Plasmodium species for structural comparison. TRAgs were selected based on having the lowest sequence identity to the TRAg domain of PVP01_0000100 CTD and were modelled with Alphafold. The structures were superposed with the experimentally determined structure of PVP01_0000100 CTD and the RMSD values were based on the number of residues aligned as shown in the table (*bottom*).

Supplementary Fig. 11

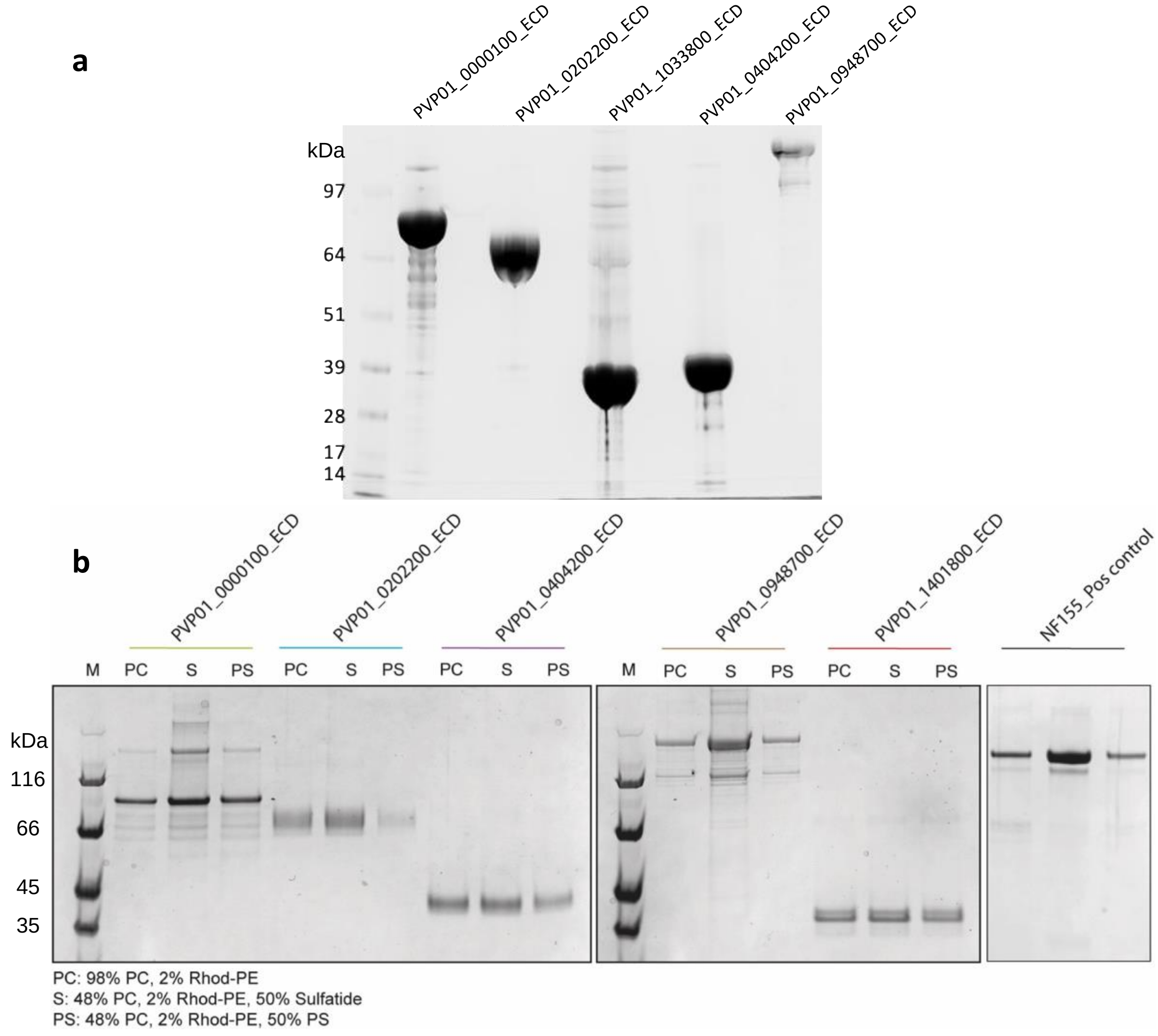

Supplementary Fig. 11. Lipid binding properties of additional PvTRAg ECDs. a) The chosen five PvTRAgs ectodomains were affinity purified followed by size exclusion chromatography. **b)** The purified proteins were incubated with liposomes composed of phosphatidylcholine (PC), Rhodamine-phosphatidylethanolamine (Rhod-PE), and either phosphatidylserine (PS) or sulfatide, and analysed by SDS-PAGE with Coomassie staining. PC liposomes are 98% PC and 2% Rhod-PE, S liposomes are 48% PC, 2% Rhod-PE and 50% sulfatide, and PS liposomes 48% PC, 2% Rhod-PE and 50% PS. PVP01_0000100 and PVP01_0948700 have shown preference for the sulfatide-containing liposomes. PVP01_0202200, PVP01_0404200 and PVP01_1401800 have shown lipid binding but without any clear preference for the lipid composition. NF155 was used as positive control for sulfatide binding.

Supplementary Fig. 12

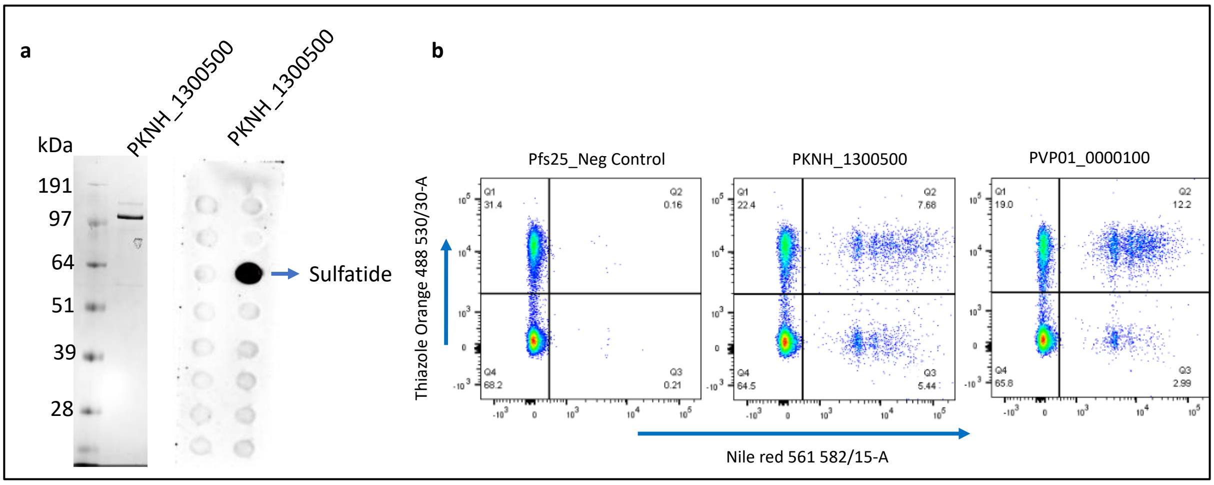

Supplementary Fig. 12: a, Highly pure homogeneous protein band of PKNH_1300500 near ~97kDa in a 4-12% SDS PAGE. The purified PKNH_1300500 has shown a specific and clear specificity towards sulfatide. This data repeated two times independently with same observation. **b,** Red Blood Cell binding assay showing PVP01_0000100 and its orthologue PKNH_1300500 showing binding towards the retics. N=2 independent experiment with n=2 technical replicate.

Supplementary Fig. 13

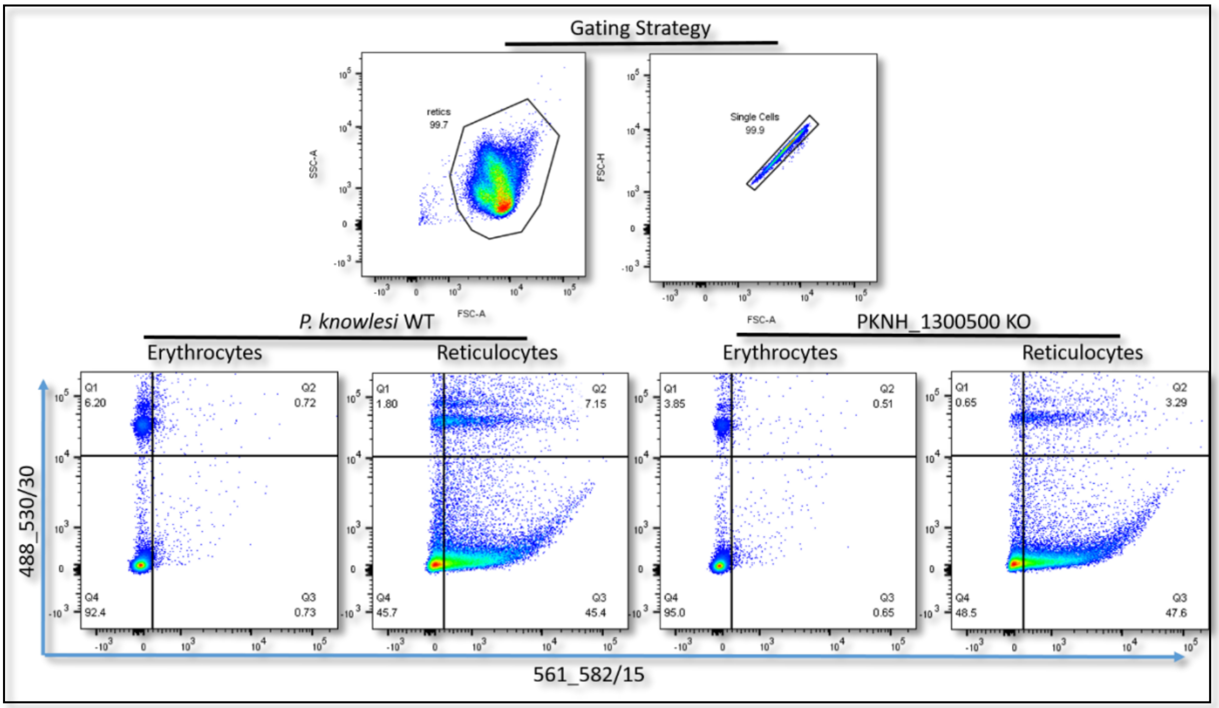

Supplementary Fig. 13: Pure population of reticulocytes was obtained using anti $CD71^+$ Ab coated magnetic beads (Supplementary Fig. 6). Reticulocytes were incubated with late parasite schizonts for 12 hours. The reticulocytes were stained with anti CD71-PE Ab (Ex. 561 Em. 582/15) and the parasitaemia was measured by staining the RNAse treated retics with SYBR green (Ex. 488nm Em. 530/30). The gates were set up based on the population of mature erythrocytes that known to be $CD71^-$ and are not get stained with anti CD71-PE Abs compared to immature reticulocytes that are $CD71^+$.

Supplementary Fig. 14

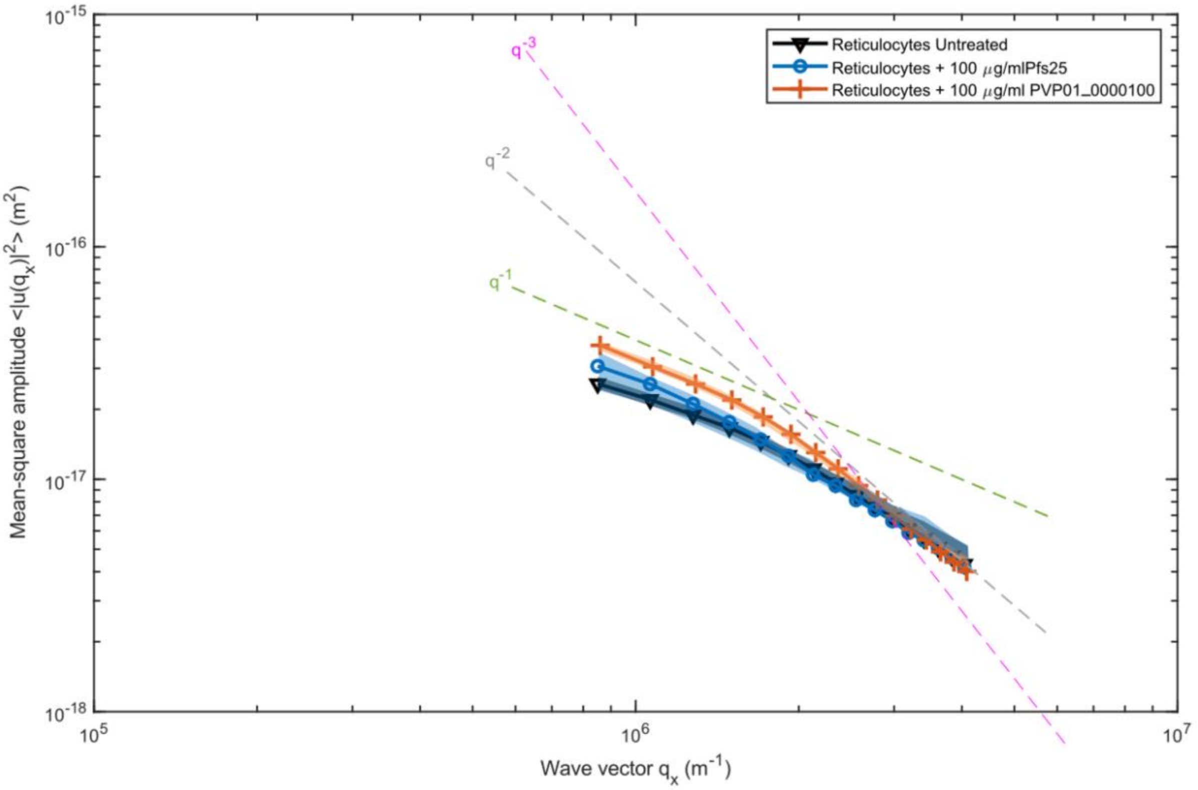

Supplementary Fig.14: Fluctuation spectra across conditions for modes 5-20. Average spectra (line) and standard deviation (shadowed band) of 65, 73, and 79 cells are shown, respectively. For modes 5- 10 tension dominates, indicated by q^{-1} dashed line, modes 11-20 tend to follow q^{-2} trend.

Supplementary Table 1: List of TRAg gene family members across *Plasmodium vivax*, *Plasmodium knowlesi* and *Plasmodium cynomolgi*. The table listed all PvTRAgs gene family members showing information regarding the level of expression in HEK cells, red blood cell binding, prediction of signal peptides, orthologues in *Plasmodium knowlesi* (Pk) and *Plasmodium cynomolgi* (Pc) and their sequence identity with Pv. The table also highlights two different naming system used to identify the members of the family in previous literatures.

PvP01 Gene ID	PvSalI Gene ID	Description	Region Expressed	Expression Trial	Mol Wt. kDa (full length)	Retics/RBC	SP (Y/N) (SignalP)	SP (Y/N)/ TMD (No.) (Phobius)	SP (Y/N) PrediSi	*P. knowlesi*	*P. cynomolgi*	Annotation - Sharma	Annotation - Han	% aa Identity Pk	% aa Identity PcM
**PVP01_0000130**[#]	PVX_112670	tryptophan-rich protein	T35- K339	Y**	38.8	Below Background	Y	Y	N	PKNH_1300800	PcyM_0013100	PvTrag38.5	TRAG28	75.25	84.85
**PVP01_1033800**[#]	PVX_097577	tryptophan-rich protein	A60- S315	Y***	38.7	Below Background	Y	Y	Y	PKNH_1032500	PcyM_1034500	PvTrag38.7	TRAG17	83.1	89.64
**PVP01_0949200**[#]	PVX_109280	tryptophan-rich protein	P22-Q283	Y**	35.26	Low	Y	Y	Y	PKNH_0400500 (non-syntenic)	PcyM_0953800	-	TRAG36	73.74	82.98
**PVP01_0948700**[#]	PVX_092990	tryptophan-rich protein	S22-1386V	Y**	154.56	Below Background	Y	Y	Y	PKNH_1356500 (non-syntenic)	PcyM_0953300	PvTrag157	TRAG15	36.26	75.11
**PVP01_0404200**[#]	PVX_002500	tryptophan-rich protein	D20-276N	Y***	33.68	Below Background	Y	Y	N	PKNH_0400600	PcyM_0401500	PvTRAg33.6	TRAG3	80.07	87.68
**PVP01_1401800**[#]	PVX_121897	tryptophan-rich protein	Y16-275N	Y***	33.05	Below Background	Y	Y	N	No orthologue	PcyM_1402300	PvTRAg33.5	TRAG21	No orthologue	81.82
**PVP01_0700900**[#]	NONE	tryptophan-rich protein	E50-298S	Y**	37	Low	Y	Y	Y	PKNH_0700400	PcyM_0700300	-	N/A	76.51	86.24
PVP01_0700700	PVX_125728	tryptophan-rich protein	N33-279S	Y*	35	Not able to test due to low expression	Y	Y	Y	PKNH_0700300	PcyM_0700200	PvTRAg34.9	TRAG34	75.99	82.44
**PVP01_1470100**[#]	PVX_101525	tryptophan-rich protein	D34-G284	Y **	35.7	Below Background	Y	Y	N	No orthologue	PcyM_0050900	PvTrag35.7	TRAG24	No orthologue	23.86
**PVP01_0503600**[#]	PVX_088820	tryptophan-rich protein	T72-316V	Y**	38	Below Background	N	Y	Y	PKNH_1324800	PcyM_0502100	-	TRAG38	80.7	87.03
PVP01_0504200	PVX_088850	tryptophan-rich protein	K65-457V	Y*	56.2	Not able to test due to low expression	N	Y	Y	PKNH_1324300	PcyM_0502700	PvTRAg56.2	TRAG7	63.97	78.88
**PVP01_0532700**[#]	NONE	tryptophan-rich protein	Q67-326N	Y**	39.97	Below Background	N	TMD (1)	N	PKNH_0516100	PcyM_0534100	-	N/A	75.77	82.82
**PVP01_1201800**[#]	PVX_083550	tryptophan-rich protein	K60-612S	Y ***	73.42	Below Background	N	TMD (1)	N	PKNH_1248000	PcyM_1203000	PvTRAg73.4	TRAG20	76.8	84.29
**PVP01_0202200**[#]	PVX_096995	tryptophan-rich protein	Q59-A480	Y *****	54.8	Below Background	N	TMD (1)	Y	PKNH_0200900	PcyM_0203500	PvTRAg55	TRAG2	56	72.51
**PVP01_1469800**[#]	PVX_101510	tryptophan-rich protein	Y56-Y715	Y **	82.28	Below Background	N	TMD (1)	N	PKNH_1472400	PcyM_1476800	PvTRAg74	TRAG22	64.57	77.84
**PVP01_1101400**[#]	PVX_115465	tryptophan-rich protein	E334-S598	Y ***	70.63	Below Background	N	TMD (1)	N	PKNH_1100600	PcyM_1100400	PVTRAg69.4	TRAG19	50.62	74.37
**PVP01_0000100**[#]	PVX_112655	tryptophan-rich protein	K62-L693	Y ***	80.8	Y (High)	N	TMD (1)	N	PKNH_1300500	PcyM_0013400	N/A	TRAG25	68.42	85.69
**PVP01_0532900**[#]	PVX_090265	tryptophan-rich protein	E63-K326	Y **	40.1	Below Background	N	TMD (1)	Y	PKNH_0516300	PcyM_0534300	PVTRAg	TRAG11	70.25	80.67
**PVP01_0503700**[#]	PVX_088825	tryptophan-rich protein	L60-S444	Y **	53.74	Y (Med)	N	TMD (1)	Y	PKNH_1324700	PcyM_0502200	PvTRAg53.7	TRAG6	73.41	82.61
**PVP01_1469900**[#]	PVX_101515	tryptophan-rich protein	K61-S321	Y **	39.93	Below Background	N	TMD (1)	N	PKNH_1472500	PcyM_1476900	PVTRAg40	TRAG23	67.29	82.55
PVP01_0532800	PVX_090260	tryptophan-rich protein	D68-346V	Y *	42.92	Not able to test due to low expression	N	TMD (1)	N	PKNH_0516200	PcyM_0534200	PvTRAg42.9	TRAG10	58.59	75.54
PVP01_0801800	PVX_094305	tryptophan-rich protein	R41-N797	Y*	91.57	Not able to test due to low expression	N	TMD (1)	N	PKNH_0800900	-	PvTRAg99.6	TRAG14	60.64	63.87
PVP01_0000110	PVX_112660	tryptophan-rich protein	D64-K312	Y *	37.6	Not able to test due to low expression	N	TMD (1)	Y	PKNH_1300600	PcyM_0013300	PvTrag26.3	TRAG26	63.34	79.81
PVP01_0503400	PVX_088810	tryptophan-rich protein	N61-E358	Y*	42.94	Not able to test due to low expression	N	TMD (1)	Y	No orthologue	PcyM_0501900	PvTRAg32.4	TRAG4	No orthologue	86.02
PVP01_0533100	PVX_090275	tryptophan-rich protein	A25-V339	Failed	38.8	Not Expressed	Y	Y	Y	-	PcyM_0534400	PvTRAg38.8	TRAG13	No orthologue	79.29
PVP01_0000170	PVX_112690	tryptophan-rich protein	P33-313K	Failed	36.63	Not Expressed	Y	Y	Y	No orthologue	No orthologue	PvTRAg36.6	TRAG32	No orthologue	No orthologue

PVP01_0000120	PVX_112665	tryptophan-rich protein	A29-288L	Failed	35.24	Not Expressed	Y	Y	Y	PKNH_1300700	PcyM_0013200	PvTRAg 80.6/Pv TRAg35 .2a	TRAG27	60.07	77.78
PVP01_0949000	NONE	tryptophan-rich protein	K42-T334	Failed	40.43	Not Expressed	Y	Y	Y	No orthologue	PcyM_0953600	N/A	N/A	No orthologue	37.3/44.34
PVP01_0948800	PVX_092995	tryptophan-rich protein	Q45-360A	Y*	43.29	Not Expressed	Y	Y	Y	No orthologue	PcyM_0953400	PvTrag4 2.9a	TRAG16	No orthologue	63.50/36.99
PVP01_0533000	PVX_090270	tryptophan-rich protein	T29-406E	Failed	48.55	Not Expressed	Y	Y	N	No orthologue	No orthologue	PvTRAg 43.1	TRAG12	No orthologue	No orthologue
PVP01_0201100	PVX_096950	tryptophan-rich protein	35P-L327	Failed	39.95	Not Expressed	Y	Y	Y	PKNH_0200500	PcyM_0202300	PvTrag3 9.8a	TRAG1	19.37	18.31
PVP01_0000200	PVX_112705	tryptophan-rich protein	P25-A362	Y*	45.25	Not able to test due to low expression	Y	Y	N	PKNH_1301000	PcyM_0012700	PvTRAg 37.4	TRAG33	64.65	84.29
TRAgs not able to Include in the retics/erythrocytes binding study as gene synthesis failed															
PVP01_0000140	PVX_109280	tryptophan-rich protein	-	-	33.4	Failed in Gene synthesis	N	N	N	No orthologue		PvTRAg 35.2	TRAG36	No orthologue	38.02
PVP01_0700800	PVX_125730	tryptophan-rich protein	-	-	40.82	Failed in Gene synthesis	N	Y	N	No orthologue	No orthologue	PvTRAg 40.8	TRAG35	No orthologue	No orthologue
PVP01_1033900	-	tryptophan-rich protein	-	-	266.13	<200 kDa	N	Y	Y	PKNH_1032600	PcyM_1034600	PvTRAg 309	TRAG18	52.41	61.59
PVP01_0948900	-	tryptophan-rich protein	-	-	39.8	Failed in Gene synthesis	Y	Y	Y	No orthologue	PcyM_0425400	-	-	-	75.79
PVP01_0000210	-	tryptophan-rich protein	-	-	33.3	Failed in Gene synthesis	N	N	N	No orthologue	PcyM_0012600	-	-	-	91.80
PVP01_0532600	-	sporozoite and liver stage tryptophan-rich protein, putative	-	-	39.83	Failed in Gene synthesis	N	Y	N	PKNH_0516000	PcyM_0534000	-	-	85.09	92.24
No orthologue	-	-	-	-	-	-	-	-	-	PKNH_0200400	None	-	-	-	-
No orthologue	-	-	-	-	-	-	-	-	-	PKNH_0118300	None	-	-	-	-
No orthologue	-	-	-	-	-	-	-	-	-	PKNH_1326300	None	-	-	-	-
TRAgs not included in the study due to low score (E value cut off <0.01 and Bit score >40) in Blast with C-terminal Tryptophan Rich domain of PVP01_0000100															
PVP01_0000160	None	tryptophan-rich protein	-	-	31.7	Not Included in the study	N	N	N	No orthologue	No orthologue	PvTRAg 34	TRAG31	No orthologue	No orthologue
PVP01_0000150	PVX_112680	tryptophan-rich protein	P33-K313	Y*	36.75	Not Included in the study	Y	Y	Y	No orthologue	No orthologue	PvTRAg 36.7	TRAG30	No orthologue	No orthologue
PVP01_1301100	PVX_084115	cytochrome c oxidase assembly protein COX19, putative	-	-	24.78	Not Included in the study	N	N	N	PKNH_1301700	PcyM_1301600	-	-	87.68	91.94
PVP01_1102700	PVX_115425	conserved Plasmodium protein, unknown function	-	-	50.15	Not Included in the study	N	N	N	PKNH_1101900	PcyM_1101800	-	-	66.04	79.40
PVP01_0002460	None	Plasmodium exported protein, unknown function	-	-	29	Not Included in the study	N	TMD (3)	N	PKNH_1247800	None	-	-	25.48	-

A total of 18 genes, highlighted in bold, were used in the red blood cell binding study.

Supplementary Table 2: Signal peptide prediction of *Plasmodium vivax* TRAgs Signal peptide prediction by three different servers against 38 PvTRAgs were performed. The number in each column indicates the number of genes predicted to have signal peptide as an output from each server Signal peptide prediction by three different servers against 38 PvTRAgs were performed. The number in each column indicates the number of genes predicted to have signal peptide as an output from each server.

TRAgs (Total)	SignalP	Phobius	PrediSi
38	18	23	20

Supplementary Table 3: Sequence of the constructs used in this study.

PVP01_0000130<br><br>AAATEGGDNLDDDLGGDLEGLLGDDAGEGGAAGGEGAAEGAAAAASAEGLSGEVENELLYVKEDDDAPAATPDEKPSTSGEETPAAFVDLV<br>NEAVPPPAKAPLPLQTKAPQGPKIKDWNQWMKQAKKDFSGYKGTMHTQRHEWTKEKEDELQKFCKYLEKRWMNYAGNIDRECRSDFLKST<br>QNWNEAQWNKWVKSEGKHHMNKQFQKWLDYNKYKLQDWTNTEWNKWKTTVKEQLDDEEWKKKEAAGKTKEWIKCTDKMEKKCLKKTKK<br>HCKNWEKKANSAFKKWEGDFTKKWTSNKQWNSWCKELEKGAP
PVP01_1033800<br><br>AAAASTERGSIKCYLPNNLMEFWKDDVDQSEQLKKCAWNNWMMRLESEWENFNTAMKSKKNVWLQETEQEWTEWIKQMENKWMNCNENI<br>NDEYKDYLISKSATWTDEEWKEWIKTEGKNFMKTDLEKWIKAKETSLDLLLLTEWVQWKNEKIMAWLLSEWKTEEDTYWSQWEHSTWLKWL<br>NLAEKKHWLKWKERNHREGEQWSTWLHVKENVYIFSEWNNWAIWKNEKEEFFYKWMKDTINEWINEKRWNTLVSTDNDAGAP
PVP01_0949200<br><br>AAAPASLQNECNNAKDCALNIANEETAEIEKTDEWKENEWNKWKLKLEDNWKQFNLALIKEKNEWIQNVAKDWNEWMQN MQHKWTHYIEN<br>MDESHKSHILEKSLTWNNADWENWVNTELKGLIDNEWQNWINQIESQRYTWIEDKWVHWRSYQILTWLKSDWKHDENICWLRKEYIEWNKP<br>SKVRNTGGWLKWKERIYKQSLEWLYWVQNKEKIIQNIKCSGWIRWKDDKNKIFNQWKECFLNNWIREEKWNSLFNDKQTLFLRQGAP
PVP01_0948700<br><br>STNDLELENAADDVVEVEDPSNDGLELEEENFDENSGDDETLLDATPEDDFALTDLPIEDDEEVNEALDGGESLGEVSTEDMETEDGSTDDTE<br>TEEGLPGDMEGEEEAGDMEAGEEAGDLEAGEETGDLEAGEETGDLEAGEEAGDLEAGEETGDLEAGEETGDAETEEGATGDAETENGATV<br>YVDTEDSSADGAEKVHVPAQENVQPADSNDALFGSILDKDIIFDHIKDFEPLFEQIVAGTAKHVTGQELPMKPVPLPVAEEPAQVPAEELDATPE<br>DDFALDVTESPEEVELVLDEEATEEESTEVGPTEEGPTEELDATPEDDFALDETAEGETEETVEGEETEEAAEGEVSEETPEGEEELEATPED<br>DFALDGTALEETEETVEGEETEEAAEGEETEETAEGEVSEETPEVEEELEATPEDDFALDETLGEETEETVEGEETAEGEETVEGEETVEGEE<br>TVEGEEELEATPEDDFALDGTTLEETEETAEGEETVEGEETVEGEEAAEGEEELEATPEDDFQLEEPSGEGEGEGEGEGEGEALVAVPVVAE<br>PVEVVTPAQPVKPMVAPTADETLFVDILDNDLTYADITSFVPLFKQILKDPDAGEDVTVPSKEAPVQVPAVEPAQEVPTEELDATPEDDFELEG<br>TAEAPEEGELVLEGEGEPTEEEPTEGEPTEGEVPEEELEATPEDDFELEEPTGEEVEETVEGEETAEGEEELEATPEDDFQLEEPTGEEVEVE<br>VEEPAEEVEAEGEPGEEEVQEEVPEEELEATPEDDFQLEEPTGEEVEVQEEEAVEEEEAVEEGVEVEGEPGEEEVQEEEVVEEGVEVEGEP<br>AEEEVQEEEVVEGVEVEGEPAEEEVQEEEVVEEVEAEEPVEEKPKVEELKPVETPKEAKLAVPADTKTELTVAGPMSYDALFALIVDGDLTHE

AIKRYEDVFKQLLKDPEARRLLEQQQQQPQPQPQPQKPVEVPQEVEEVEEVPAEVEEVEEVPAEVEEVEEVPEEVEEVEEVPAEVEEVEEVP<br>EEVEEVPEEVEEVEEVEEVEVPAVVEVEVPAVVEEEVPEEVEEEEEEEEPVEEEDVLQLVIPSEEDIQLDKPKKDELGSGILSILDIHYQDVPKEF<br>MEEEEEETAVVYPLKPEDFAKEDSQSTEWLTFIQGLEGDWERLEVSLNKARERWMEQRNKEWAGWLRLIENKWSEYSQISTKGKDPAGLRK<br>REWSDEKWKKWFKAEVKSQIDSHLKKWMNDAHSNLFKILVKDMSQFENKKTKEWLMNHWKKNERGYGSESFEVMTTSKLLNVAKSREWYR<br>ANPNINRERRELMKWFLLKENEYLGQEWKKWTHWKKVKFFVFNSMCTTFSGKRLTKEEWNQFVNEIKV
PVP01_0404200<br><br>AAADISPSLQYEQISPTPDLTNPVQSEQECAANNVILREEQNDNWMVKLEGEWKDFTASLENEKIRWIQEKEKEWDEWLEIMQEKWTHYDRN<br>LNPAYKNYILKKSSEWDNFDWEYWANTEWHELMEKDWKNWIYGNKLSLNKIIDNKWINWANEKMAEWLIQELNDEEGSDTQTTNIGKSSSSE<br>ENDEYFSSLNDKIFSKNEEWEHWTDRKEKLIKKIKNSNWAEWKNNKYASFNQWRESFIKKWLRERQWEIMVNNQINGAP
PVP01_1401800<br><br>AAAYLLSNIAPSSQSAVDYIEQEPLDILNLEEGDLEVTEQWKDNEWHNWKLKLEEDWDSFSTSLIRDKKDFMKIKTDELNGWLNLEENKWNNF<br>AGYLSDGYKNYLLKKSEKWNDADWENWANTEMVAHLDKDYHLWSLNTERSVNALVRGEWNQWQHDKMSSWLSSDWKKVGAMYWDLQE<br>SRNWASYSHTDDMKEHWIKWNDRNARENIEWSKWVQNKEYFIMYARHSDIEQWKYDNYALYSTWRNDFINRWVSEKKWNSILNGAP
PVP01_0700900<br><br>AAAENPQEPKERSDEWRQNEWNKWEDKIEEEWAEFDENLEAEKDELIKMNEKEWGPWFESIQKKWTHFGKNIDDTYKSEVLKGSLYWDEA<br>SWRTWIKTEGRAFIHRDLAKWFASKESYLYRWVSNQWTEWRSEKIQEWFNSKWKREENDYWEKWEKKFKPSQNSAKMVNSKDYKKWFKW<br>KERTQLELNQWHEWAEFKDYIYTNSAWAKWKQWKNAKRTLFYDWVDSYVYKWIRENQWTVLVDEIKELAPRKSGAP
PVP01_0700700<br><br>AAANEIERLTHVKLKDTSEWTENVEEWVKDEWHEWMDEVQMDWKEFNSALESEKNKWFGKKEKEMMELIKSIEDKWLDFNENMHEVLNYAI<br>LKISLMWSFSEWQKWINKDGKRIIENQWERWTISNKNLYYKIIMKEWFKWKNKKIKQWLKRNWLHHEGRILENWERLPYTKILAMSEKKPWFN<br>SNAQVINERDYFLIWIKKKEDFLVNEERDKWENWEYYKNDFFQTWMDSFLSHWLNIKKRDILHSQSGAP
PVP01_1470100<br><br>AAADEVEDEHIVNLDDIETIQGHSSELDNEANDQLNEEWGEWQLTFEEEFQNFLLSLENNAADMLQEEKNEWDYWIEKMQNKWSRFHGIMSE<br>SYKSYIYNKAATWTEADWKTWIHTDWKDYMEIDWHRWINDVEYDLDKLIDERWEQWKTHQIMTWLMSNWADEEQQYIDKLEFGKYSNKKEM<br>EHFVKRWNKIIQWKTEKWESWIQDKEGFITNIKNEKWLQWKDDHYFLFNNWRNDFIEDWISKKRWLLWSAGGAP

PVP01_0503600<br><br>AAATLIGFGELSIQESEEFKRMAWNNWMLRLESDWKHFNDAVEEAKTKWLHERDSAWSDWLRSLQSKWSHYSEKMLKEHKSNVMEKSAN<br>WNDAQWGNWIKTEGRKILEAQWEKWIKKGDDQLQKLILDKWVQWKNDKIRSWLSSEWKTEEDYYWANVERATTAKWLQEAEKMHWLKWK<br>ERINRESEQWVNWVQMKESVYINVEWKKWPKWKNDKKILFNKWSTNLVYKWTLKKQWNVWIKEANTAPQVGAP
PVP01_0504200<br><br>AAAKKSNRLSLLWNRKQEDNENEEEETEGEEVADQEEAVKSDEDEDEETFYDSSDKEVYLTEQEEKDKENEMYLTESEGRKKIRKLISEADK<br>QDNQAVMSYLAELPEEELEEWKNQEWKKYMENIEEEWQLLNLWIEEEKQNWIESKDKELENWMNKMENKCMHIDNINKEYQCMFIKSCLKG<br>DDQSQIKEQLKHELKNFIYRDWKKWLRENEAYLNTWLVKQWIQWKNNKILKFLMAEWKQEEDEYWNDWEKTKTWKWLHFYKRRKWQTWK<br>NRVTSEKQEWENWVKIKEERVIYNKYKKWTQWINAKKPSINQWVESLADKCVNDARWNTWIDEKYNEFLLEQKMEKKEKRKEKKNLMKKFK<br>NKKNNLAFAFEFFENNLQSIKEEEEEGENALSVGAP
PVP01_0532700<br><br>AAAQKDEPPKEVTNKEMEEENTKTDEWKAKEWKAWMEQLDKDAEIFTTSLEHKKDQWLVQRELEWGDWVKSMEEKWNHYNEKMGSEYYS<br>FIYKNTAAWSDEQWENWINTEAKQLMEIDWSDWISESESYVDVMMVKEWIVWKNNKIMEWIMRDWKCKEDEQWDAWEKNKWSKWFSINER<br>KKWTQWKDRLSKETEEWTAWVENKEKQYLDNEDRKLVEWKKNNYILFNKWMESFINKWIKEKQWKHYSKQPTGVEPPAAMSLSNGAP
PVP01_1201800<br><br>AAAKKMKILQPRENHEIFYDALDTLDGDLSTEEKIQKQLREVENHNRKVQKLQDWFKDVEKRVKAERRKIDLATGRAKSDDLIKRARQTSKYVI<br>KPEQEIALEDTEEKEIMENGVKKIQNGLRIIEELMQKLDDGTGKMPKDNLGSYKQEVEIPEDKIAEFDEGLELFQEGLKLIEARVKNNAGDNEIEE<br>IKIELEEPNEEEEDGDNSAPYLYSQDAVVQEPLLASERKSYKGKMAQVEKWINEIDETIKNEAMKIKEGVEAEVKPPESFAQDAGKTKGQAEKN<br>TAKEETHENVYEQGQNMGEEPSGELTEEWKAKEWKKWINNAEKQWGNFAQSFEQHKQKWVNKKESEWEEWLTNIHYNWIGFTNKLEGDYI<br>NDKVNAWTKWGEKEWKGIIEMEWKRPMKVKWTKLVEKNEKIWGDKLFNYWDNWKDKKWNEWKNMNWKKKEEEKWNNAEKRKDLNKDEN<br>WKEWKKRLLREKKEWENWVNKKEDFLMDSEETHWEKWKEYKWNYLNEWMKQVEVDWLKSKPWEVWKQARSDFYESNVKLEDDSQHSE<br>GEYSVSSGAP
PVP01_0202200<br><br>AAAKKMKILQPRENHEIFYDALDTLDGDLSTEEKIQKQLREVENHNRKVQKLQDWFKDVEKRVKAERRKIDLATGRAKSDDLIKRARQTSKYVI<br>KPEQEIALEDTEEKEIMENGVKKIQNGLRIIEELMQKLDDGTGKMPKDNLGSYKQEVEIPEDKIAEFDEGLELFQEGLKLIEARVKNNAGDNEIEE<br>IKIELEEPNEEEEDGDNSAPYLYSQDAVVQEPLLASERKSYKGKMAQVEKWINEIDETIKNEAMKIKEGVEAEVKPPESFAQDAGKTKGQAEKN<br>TAKEETHENVYEQGQNMGEEPSGELTEEWKAKEWKKWINNAEKQWGNFAQSFEQHKQKWVNKKESEWEEWLTNIHYNWIGFTNKLEGDYI<br>NDKVNAWTKWGEKEWKGIIEMEWKRPMKVKWTKLVEKNEKIWGDKLFNYWDNWKDKKWNEWKNMNWKKKEEEKWNNAEKRKDLNKDEN

WKEWKKRLLREKKEWENWVNKKEDFLMDSEETHWEKWKEYKWNYLNEWMKQVEVDWLKSKPWEVWKQARSDFYESNVKLEDDSQHSE<br>GEYSVSSGAP
PVP01_1469800<br><br>AAAYYPPMLQHAKEIYILMKHDLNNGKEEREEFMNKVTGYLRTCRVYILSHLSTILGATIKKLKSTDEGVQNVQGDATQDNLLSACHDDEDYEN<br>GDDEADEDVDFEDNEDDGEEHDENENDAMEKGSNDGSLTKLNIDMKGEGSNDALAKVDLENAPENKPNDAFTKEGEEDMTKEKTDEKFMK<br>DTEDANKQGTADNDVKEDIIEKKNEKDSITNEQVKHGENVAIESETKGQSKSETGEELKREKGGPSKSATGEATKRATGGAAKRATGGAAKRA<br>TGGAAKRATGGAAKRTTGGAAKRTTGGVAKSDASGVAKSDASGVAKSDASGVAKSDASAVAKSDASGEAKRDTTGVPKRETAGQEKLQTN<br>GQTNDKTYEELMNMAQDTNAGTNNDITKKILNFKNIISKDKENGEKGVNVDESNKELTEEWKVNEWNKWMRQLEEQWHFYFITLETKTIDWM<br>KQKEEEFNTWLTEMENKWMSYNHNLDTEYNTNLYKKYLTWDETDWKTWIKTVGKRLIEEDWVKWINDHECKLNEWFNGDWNQWKSLKNFN<br>WEMNEWKSDEYERWAEWQNGNLALWLNKKKKKKYLTWKNRIEREKSEWDSWVRAKDDLTLKSKTCKWIKWKNEKRLLFNDWVENFINTWI<br>SRKQWNSWVIERRNLLSRRTSSSYGAP
PVP01_1101400<br><br>AAAERQQESVYAKQDPFLTNKGENFYEPTPDSTERWKENEWYMWKKKLNSAWEEFNSAVENGKRKWLVNKDKDWEDWLKVMEKKWMHY<br>NERMEREYKSDLFERSADWDDQQWEEWIRTEGEQFMELDWKKWMYENKFLLDDWASKEWAKWKKDKIMSWLLSDWKLTEQKYWDDWE<br>NKAWPRFVYLRDRENWMRWKERINREWEEWTSWVQDKDKVFVNNKWVGWSKWRDDRRALFDDWMKPFIEKWINQKQWKVWKEERDYA<br>LRRNNAGAP
PVP01_0000100<br><br>AAAKKLNNALYSNTLNAIKYSQGYIDKPYEKGNTELKNEGGAFEGALAEVRTNEKEIKTGGDSISVEEATNIDKEKEGEKGKGDNKIKESEPNVA<br>NIATNYNIEKNGDVNNEAKKGAYHISTVEGKDLNKKEKSDNKAEGEKYNLMPENNDAVVNLGGTYFLEKRGMHIYDIKEEDEKLNHNTKDDKD<br>LTEAKGNADNKAASSMNVKNGANHVEKGAEASTGKKEEGEKHVDKKEPNNYQIKNEGQENDAAGTSYSLKKGADGHNDKKKSSSNSLEGV<br>QEKNERETNSHKLKEDEENVRDGSTSSLSKWGEKDEQKTVPNNDKTELDTKKVKKEDAKKDEAKKDDAKKDDAKKEAKKSDLKKDGKKEPK<br>KEKDKSKDIRRANSLDTIYKSDLESKDDEIEDDKSDEWKKNEWNNWLIKTEEDWKLFNTAVENKKNRWLEKRDKELEVWLMNMQNRWLHYR<br>ENEENEYKAEAMKNSATWDDSQWEQWIKTEGKKGMEADLKKWLNDKETFLDGWISKEWVQWKNERMLQWLSVDWKHKEDETFEHYKSSK<br>FTNVLHIKKKKKWTKWKERTNKEKEEWNNWVKGKENLYVNNKWDKWLKWKKDKRALYSQKFLTFINKWISDKQWTVWIEDQGGSTLGAP
PVP01_0532900<br><br>AAAEDFLGVESDEESEKKKELVEKSEEWKRKEWSNWLKKLEQDWKVFNEKLQNEKKTFLEEKEEDWNTWIKSVEKKWTHFNPNMDKEFHT<br>NMMRRSINWAESQWREWIQTEGRLYLDIEWKKWFFENQARLDELIVKKWIQWKKDKIINWLMSDWKRAEQEHWEEFEEKSWSSKFFQIFEK<br>RNYEDFKDRVSDEWEDWFEWVKRKDNIFITNVLDQWIKWKEEKNLLYNNWADTFVTNWINKKQWVVWVNERRNLAAKAKAALNKKKGAP

PVP01_0503700<br><br>AAALPTLGNKFKRSARNAIGYSTVEKEDVKKDAKKDGKELPSRSASKTESKENNEKNNAQKNEARKNSEPKQPNDNNVKRINKNEEKRKQRY<br>NIGLKGDEEEVQTTQSNGDGQKSQTSLLDKIINAKKMGKGGMKKLIKKPTVLHTANYNYKDSTEDLQEEKSEEWENNQWNIWKKKNEEEWKI<br>FNTAIENEKDTWLQGIEKEWQELLEAMQNKWIHYNKKMDAEYQINILEKSSQWDDTQWVEWIKTEGKQFMEQQWKMWLAQKEAHLNNWVV<br>NEWIQWKNSQIMEWLTTDWRLQKEASWSNYENNKITNMLQIKKRKQWNKWKERINREREEWDAWVRSKENIYINTKWNKWSKWKKDKRFIF<br>SKWVEMFTNKLISERQWKEWVKSGAP
PVP01_1469900<br><br>AAAKKNDNIPQLPQKKESHKEIALEQGLIEKSEELKKYAWNNWFMKLQADWKYFNSALENEKQTWFDEKEKEWQEWLKSMENRWTHYNEN<br>MDAKFKSYILKNSQGWDDNQWETWIKNDGKKFMETNYHKWIDENYANYNAWVVKRWEQWKNEKILTWLLKDWRRNEFEYWYKFKNMALP<br>EPLYERAKNNWNKWNKRLSKEKEQWKKWVAAKYELYENSECKQWKKWKDDREVLFNNWIESFINTWVAEKKWNVWIEEKKNAATSGAP
PVP01_0532800<br><br>AAADMYDSIGAVYFDQKLIKKSEELKRYAWENWMARLELDWKHFDNSIKSKKEKWLEERVHSWEEWLKQIEDKWEHYNAHMDMSYKFYIFKI<br>SSAWNQAHWEQWAKAELKYFIDREWHNWVYQNEQHLNNWITNEWSEWKNHIIAAWLTKNWKLSENAYWEKMAKKKWVKSLFRVVRKNWL<br>KWKERIDREFQQWDEWVVGKQHLYTNNHEWDAWVKWKTDKYALVKEVRKSFVTKWVTEKQWRAWVEERHNLIQQEKYRMGIWGDPQMG<br>RTVPTWGGGPSTVGAP
PVP01_0801800<br><br>AAARRYYDSSYTDFEEIKKSDTSENSANDKDYNQANNIVDQIINPNFANIKIGSNSNIADHIVSHARGEYYPNIANEKFTKIDDETFSNLLDGSLSK<br>FENENFAHATEHKFEKLPTLKFVKQTTSKFSKLITPCQRNTATDTCPDVSNEQLFNLMSQKAPNDESERAPNDESERAPNGESKKAPNDESKK<br>APNGESKKAPNNEAKKAPSDETKKAPSDESKKSPNGETKKTPSDESKKSPNGESKKTPTTITKKRTSDESKKPAHVTSKKPPNVESKKPPTVK<br>SKKNPNAKSKQSPSAATKKSPNTGTKTPPSAATKKSPNTGKKTPPNAATKKSPNVATKRTLSIEAKKATNEIFKEDPNLGANKAPAQTAKKAPA<br>QTAKKATEVTTKKTPTKAAKKTTEETTKKTPTETAKKPPNAANEKKGTPNVDEVEPAAKSYFQNFMDNTVFAWREKIHSKMNANKEKLLQDKT<br>TQSSEGEQGDIKDGSKKPKKEEPTEEWKIQQFKNWLNNLEKEYDAWKVSLVNDNNEWILKKNEIFENILNRIKEKWESWNIYTLKDINDDVIRL<br>KDLDTEEQWSKWLDAQWKPYNKQTWFDLIDSYEKLYHQWKRTHWDKWKSRKMTEWISQEWKVDEEEKWAQWTSQKWEKYFQRKEKKE<br>WIKWIERNEIEISIIRDWLKEKESMPVQCDGWLEWDTWKQEKFQHLDDYLNSLKNQWLAEKKWMILANAAKERELAEQRAAPGGKKAAAKGA<br>PQNGDKADDKADDKADNGAP
PVP01_0000110

AAADKYDAVAKPACLVGLVEEGPGEEEEWKRYTWDNWKTRLEEDFNEFYFSLDKEKKEWIEEKEGEWEKLIHEMGNKWTTPEKGFDNRAHL<br>SDSLEGSSTWNDAQWEDWIRTKGSQLMEIEWENWIGEKDSLFNEMILKKWIQWKNSKIMSWVTSEWKTEEDYYWANWEKNMACKCLHLGE<br>KTKWLKWKERTSRESAEWTDWVNNKECVYIRKVWNKWTEWKNNKKTLFKIWKDLFIDKWVDKKQWLAWTLKGAP
PVP01_0503400<br><br>AAANPLSGVLQNLANAASGTSVRTTALTEKLKENKWNDWMKQLEEQWKSFHDQMENERKQWLEKKDAEWEKWKKYTEIKYMSYCVDINNE<br>FESNTLKDSSTWNRNQWEDWITTVGKEMIKSEYEKWIYENENYLDEWKVREWVEWKQKNIASWLSSEWKCKEDDSWSKWEQTVWGKWLF<br>TENREKWIAWKERTLRERAEWLKWVQLKEYVYINDEWIPWTKWKNENRLAFIDWIGSFMNEWINNKQWNEWIHERKKMVATKKIAPLNAENA<br>ANAASSVGVRVNDALQSDAPQSACAQDEAEGAP
PVP01_0948800<br><br>AAAESQGCSGCRLPPAKRWFTFTSQPYCKTAPYYDYDLKHMPYYVNVVSESENVKHDKWDNWLKEMKISLTEKLEKESQEYMEKLEEQWD<br>EFMKNSEDKWRHYNPQMEEEYQCSVYPLGLKWDDEKWTAWFYEKGLWCLKKSFKTWLTDSKKGYNTYMKTVLQEFGKQFYEDWCRRSEK<br>LREDKIFKRWEQKGLRGDQYYSLKWMQWRNWKNRNHDQKHVWVTLMKDALKEYTGPEFKLWTEFRKEKIDFYKQWMQAFAEQWTQDKQ<br>WYTWTEERNEYMKKKKEEEAKKKAASKKKAASKKGGAAKKAPAKKAPTKKAAPGTKAPAKKAAPKKVAAPNAAGAP
PVP01_0000150<br><br>AAAPKPDQKNLKGGVKNAPLQQRKGSVPINPPKPVNDKLKDGSNKAETKNAKNTLSKPPMQVTDKSKDEAKKTPLQSTPKLTPKTKEVPKES<br>NMEMWLKDTKDEYENLKCQYRTCLYDWFRKINDEYNELLNKLEEKWAKFPNDPKNKDVFDNLKTSSLKNDEKKAQWMRKNLKDLMREQVD<br>EWLEGKKKIYEGMSPTYWDAWEKKIAKGLMGAAWYKMNSAGRTKEWDKLRNELETRYNKKIKSLWGGFHRDVYFRFKEWIEEVFNKWIENK<br>QIDTWMNSGKKGAP
PVP01_0000200<br><br>AAAPALNEYFQKYKTKMKTRGYQESTSLQNSEFRDRPEKWKEDQWNDFMKETERDWEKFNTAMENLASSWFEKKELEWEGWIKAMQNRW<br>AYYNKNMDDCVLNVIKNSLNWADFQWQKWIRLMKNKAMKHFESSGDEYILDVFRRNTAWTTEQWKEWIKTPIRESMEKDWEYWIAEDQYKL<br>DNWMLENFDKWKTKRITEWKEKKWKAEEDEYWANWEAKGSKDKSKVLIDRKNWNLWKERTYREERQWEMYTERKKKEFFSKDVVEWMK<br>WKSEKTEMFENWKENTLSTWIKEKQWNVWLHKDKEHNWNKWCSTQKEEAGEDAWEGAREEAREGAREEALEEAGAP
PVP01_0533100<br><br>AAAAQKKAGKPKGAAATKAATAKGTAAKAATAKKPAAAKAEPSSSQEAASTPAAAAPAAAPSAAEAVKKPKAAAVITAAKPNEYVEEWRVKPL<br>EWKLNEFSNWLKKAEEDWKEFHVVLEDERTKWVANLGDLEKGWFETAEKKWNNYNPGLDLEYKSNILKKAKVWKEGQWKEWIDKSLKKYIT

DDFKKWMDGHQENLNAALSVNWETWKRQKLLECNSVGWRLKEDKHWIRWTFFSPNIDDIPVPKEFKALYENWKVRRKAETDQWKEWTDRI<br>EKRVIFKDTPAWTKWKNDKTAAFEKWLAAFTNKLVAQKQWTNWVGAP
PVP01_0000170<br><br>AAAPKFPRNNLKGGLKDSPLKQPKSPLINGPPKPVNDKLKDDSNKAETKDAKNGLNKPPKNINDKVKDGENKAPSQDLNEPSFKLPMRQKES<br>SWYTWLKGTKKDYETLKCFAKGNLYDWLCNVRESFDLYLQSLEKKWTTCSDSATTLFLCECFAESSGWNDAQWGNWMNNQLKEQLKTEAE<br>AWISTKKKDFDGLTSKYFSLWKDHRRKELDADEWKNKVSSGGLSEWEELTNKMNTRYRNNLDNMWSHFSRDLFFNFDEWAPQVLEKWIEN<br>KQWNRWVKKVRKGAP
PVP01_0000120<br><br>AAAADAQGENVHVRIPTSLKQLNEEGVYSVSEWKKKKWSEFLKSVDGDFKEFLVYLDNEKNSWLEGKDELWEQWKANMEKKWEHYDEYTF<br>KELLADHAKDALTWNDKEWINWVSKKGRLAMKGDWNKWVNGHDVFFKDGINNDWKNWADFKRKEFGSITWKYREDKFWKYWIDHGKPSE<br>PMFVIKMEMLKKWQDRLKNEEEEWSNWLDEKQKKYIDVEWDKWVQWKKENNKKFIDWMEYFVEKWTSNKQWNVWIEERNSCITLGAP
PVP01_0949000<br><br>AAAAVKKGCCYGMNPAKFNSVPLCKKPSYIRPPLTTLELEEYYRRLDVSDLEKKYEWDCFFEHQKDKIEKYLSAQMDHAVQKMEKDWDEWM<br>RKTENKWMHFNPDMQKEYNCAVYPEALKWGVTKWISWFHEKGLSCLKKDFQKYIASARKAYRDYMLSCVLEMNKAYAKEFCSRSWKLYDD<br>YVCKKWEKRGSRDSPFYVIMRLQWCQWANRNRREKEQWKNLMQEMAKKYVNVLNCPDYRDWKKDKMDFYKEWLRTFTKAWITDEQWTK<br>WTEERKQYILTKKPDTQKSAGKKPAAKKTTTKKPATKKTTTQKKNVKTGAP
PVP01_0533000<br><br>AAATNEIKNGQNGAVQLKEKGGGVNLAPKVGTNIAQKGDTKMAKKTVTKVAKKKVTKVAEKTGTKVADKTGTKVADKTGTKVAEKTGTKVAE<br>KTGTNIAQKEDEKGPPKEDTQGTQKADAKAIQQADAQVSEKWKKKEWKEWIKKAESDLDIFNALMDNEKEKKWYSEKEKEWNKWIKGVEKK<br>WMHYNKNIYVEYRSLVFWVGLKWVESQWEKWILSDGLEFLVMDWKKWIKENKANFDEWLKSEWDTWTNSQMEEWKSSNWKLNEDKRWE<br>MWENDKKWIKWLYLKDWINCAKWKKRIQKESKEWLRWTKLKEEMYKNKYGDKWKEWRTDKSIVYDDWKDDLAKRMIKKKQWQVWLKERT<br>ALIEKRKAAYPAIMKDIRFIEGAP
PVP01_0201100<br><br>AAAPPLVDIPGASSVELRSFSNQLRQYENRNSEEGHLKKWIGELNDEFNQFKDIKFKEIEKTEKQKEEAWDHWLTVLEPEWVTFSNFIEQKKK<br>RWIQKKEEDWSIWIKDMEHKWVNYKKKKDRKYLSKYRDGTLNCAEREWNVKMREVEAAMIADYKKWMRDAEENLQKWISKDLDIWKKKKF

DEWLKVEWKKEEDAHWSKDSNTPYGDTMNPLFSVLKKSYDMYTARKKREKEHWNKLTENIGKTLTNKKYVDWENMKMTKSGWYNEWMDF<br>FLQQCLNSKVRKHRSHRRRSESLGAP
PKNH_1300500<br><br>AAASVFKKLNNALDSNTLNSIKYGQGYVDDFKEKGNTELKNEDGDVFEDALEEVCTSDKGVQTDGNGIPVEEATSNDKGKENEKEKGDNQIK<br>ESEPNAASINKNYNIEKNADVNNEVKAPDYISTVEGKELNKKENSDSNNKEGEKPNIVPENNDAVVNLGGAHFSEKRGTHINNIGGKEEELNKN<br>TKDDKEITEDKGNTINKAPYSMYVKNSASHVQKAAEESTKDELGEGEKNVDKKKPSNYPIKSDGQVNQNAATSYSFKRGAGSYNNRRRYSSIS<br>LESVQETNERETDNYKFKEGRDAQYRSTSSLSKLDEKYEQVTFPDMRKMELGTRKIKKEIVKEKKEDEAKKDETIKDETKKDGTMKDAQIKGA<br>RKSDLKTDEEKDQKMGKEELKEVRRAISLDTIYKSDLEGKDEEMENDKSDEWKKNEWNNWLAKTEEDWELFNKAVEDKKNSWLEKEDEELE<br>AWINSMKNRWMHYRENEENEYITRAMRKSSIWGDRQWEQWIRTEGKRGMETDLKKWLNDKESFLNDWISKEWTQWKNERMLEWLAVDW<br>RHKEDETFENYKSSRFTNMLHMKNRKKWMKWKERTDKEKEEWNNWVRGKEHLYVINKWDKWLKWREKRALYSQKFMSFINECINKKQW<br>VVWIEDQKDSTLKKKGAP
PF3D7_1031000_Pfs25<br><br>AAAKVTVDTVCKRGFLIQMSGHLECKCENDLVLVNEETCEEKVLKCDEKTVNKPCGDFSKCIKIDGNPVSYACKCNLGYDMVNNVCIPNECKN<br>VACGNGKCILDTSNPVKTGVCSCNIGKVPNVQDQNKCSKDGETKCSLKCLKENEACKAVDGIYKCDCKDGFIIDNEASICTGAPSTSITAYKSE<br>GESAEFSFPLNLGEESLQGELRWKAEKAPSSQSWITFSLKNQKVSVQKSTSNPKFQLSETLPLTLQIPQVSLQFAGSGNLTLTLDRGILYQEVN<br>LVVMKVTQPDSNTLTCEVMGPTSPKMRLILKQENQEARVSRQEKVIQVQAPEAGVWQCLLSEGEEVKMDSKIQVLSKGLN
PVX_121920_PvRBP2b_169T-813S<br><br>AAATNTADNIDYFDISDESNYYLISQLRPHFSNIYFFDEFKRYASYHTEIKRYEDIHKTKVNSLLNEASRAIGICNRAKNTVKGLINILENPQKFKTQ<br>RESYDVKLRQYEEKKEAFRGCLLNKNRKNLDQIKKINNEIRDLLEKLKCSQDCQTNVYFDMIKIYLVDFKKMPYENYDTFIKQYKNSYLSGVDMI<br>RKIEKQIDNPVTINAIKFTQKEMGYIIDRFEYHLQKVKHSIDQVTALSDGVKPKQVTKNRLKEYYFNIGNYYSIFKFGKDSLNMLNKALIHKEKIVH<br>NLLGELFGHLEERISKLIDSEYFITESNNIISQSEETLKLAEDVYDKNTKLIEDLTLYPHLEINEFKKDYDNNVEDLRESIIYIQSYVSSIKSAYRYNVL<br>EKDSVESKQKNIPANSNAQKKVDELLSIIDSISYSNFAVAENFQKMKDYYKEIEKLKIKILQLIEAIKKYQQHVEELINKEKAVAILKEDINKIIEYIKGII<br>EKLKQLISANKDFDKIFQQVEQLINEALFNKDQFEHNKNDLHTKMKEIMHTFHERDLQQFLDNMAKFLKDQEASYQNADSKEKLDQLLTTVKAK<br>QDELKEMKCDDIPDIIDNLKKESQNVLNLKDEVINKQFENMRTEMSSSLDQMTKEYNALKSSGAP
Bio Linker His Tag<br><br>NSGSLHHILDAQKMLWNHRDRNLPPLAPLGPHHHHHH*

Supplementary Table 4. X-ray diffraction data collection and structure refinement parameters

.

Data collection	PVP01_0000100
Beamline	I04
Wavelength (Å)	0.9795
Space group	*P 21 21 21*
Cell dimensions	
a,b,c (Å)	49.20, 52.36, 102.55
α,β,γ (°)	90, 90, 90
Resolution (Å)	52.36-1.45 (1.45-1.47)
Rmerge	0.053 (4.967)
Rpim	0.023 (2.292)
CC1/2	0.999 (0.497)
I/σI	12.8 (0.2)
Completeness (%)	100 (99.4)
Multiplicity	6.6 (5.6)
Refinement	
Resolution (Å)	46.63-1.45 (1.48 - 1.45)
No. reflections	45456
Rwork/Rfree	0.225/0.253
No. atoms	
Protein	2080
Water	98
B-factors	
Protein	46.33
Water	52.25
Ramachandran	
Favoured (%)	98.7
Outliers (%)	0
r.m.s. deviations	
Bond lengths (Å)	0.003
Bond angles (°)	0.525
PDB entry	8ARL

[a]Data in parentheses relate to the highest resolution shell.

Supplementary Table 5: Primers used for PKNH_1300500 Knockout and cloning of C-terminal domain (CTD) of PVP01_0000100 in pHLSec Plasmid and the PCR programme used for amplification.

Primer Name	Sequence
eGFP FP	atggtgagcaagggcgaggagc
eGFP RP	ttacttgtacagctcgtccatgccg
eGFP_Geno_ FP	ggcagcgtgcagctcgccgaccactac
eGFP_Geno_ RP	ggtgcagatgaacttcagggtcagc
PKNH_1300500_FP_HR1_800bp	gtaaaacgacggccagtgaattcATTAATATTTATCAATGAAAGTAGG
PKNH_1300500_ RP_HR1_800bp	gctcctcgcccttgctcaccatCTTCTTTGGTTATTTTATTTATAAG
PKNH_1300500_FP_HR2_800bp	cggcatggacgagctgtacaagtaaTAAAATTATGAAAGTGGTTATATG
PKNH_1300500_ RP_HR2_800bp	gaccatgattacgccaagctTGTACATATATATCTATACATAG
PKNH_1300500_FP_Geno_Flank	GAGAATCTTATTTTGTGCGTG
PKNH_1300500_RP_Geno_Flank	GCTATAGCTATTGTTACACGTTGTAG
PKNH_1300500_FP_Geno	GGAAGAATGGAATAATTGGGTAAGGGGG
Guide RNAs used for PKNH_1300500 Knockout	
PKNH_1300500_3247-3266 F_G 2	attACAGTGGGTTGTATGGATTG
PKNH_1300500_3247-3266 R_G2	aacCAATCCATACAACCCACTGT
PKNH_1300500_2781-2800 F_G1	attAATACATAACGAGAGCCATG
PKNH_1300500_2781-2800 R_G1	aacCATGGCTCTCGTTATGTATT
Primers for PVP01_0000100_CTD cloning	
PVP01_0000100_395K-635L_ FP	CGGACCGGTgaggactggaagctgttcaacacc
PVP01_0000100_395K-635L_ RP	CGGGGTACCtaaagtagagcctccctgatcctc

PCR Programme used to amplify the homology arms and GFP

PKNH_1300500_Homology Region 1	PKNH_1300500_Homology Region 2	eGFP
95°C - 3min<br>98°C – 20sec<br>50°C – 20sec 25 Cycles<br>68°C – 1min<br>68°C – 10min<br>4°C – ∞	95°C - 3min<br>98°C – 20sec<br>50°C – 20sec 25 Cycles<br>68°C – 1min<br>68°C – 10min<br>4°C – ∞	95°C - 3min<br>98°C – 20sec<br>50°C – 20sec<br>68°C – 1min<br>25 Cycles<br>68°C – 10min<br>4°C – ∞

Gibson assembly reaction to ligate the individual amplified components and make ready for transfection in Pk

Assembly mixture	DNA Conc. (ng/µl)
Insert (HR1+HR2+eGFP) + Vector - (0.12 µl+0.25 µl+0.12 µl) +1 µl	Homology Region 1_800bp- 88
Gibson Mix (2X) - 10 µl	Homology Region 2_800bp- 85
H_20 - 8.5 µl	eGFP- 40<br>(EcoR1/Hind III) pUC19 - 70
Gibson reaction:<br>50°C – 1 hours<br>4°C – ∞	
