# Supplementary material for: The structure of a Plasmodium vivax Tryptophan Rich Antigen domain suggests a lipid binding function for a pan-Plasmodium multi-gene family
Source: Nat Commun. 2023 Sep 14;14:5703. doi: 10.1038/s41467-023-40885-8 (PMC10502043; doi:10.1038/s41467-023-40885-8)
Supplement: Supplementary file 3 — Description of Additional Supplementary Files [file 41467_2023_40885_MOESM3_ESM.pdf]

Description of Additional Supplementary Files

Supplementary Movie 1: The fluctuations and contour detection is shown for untreated reticulocytes.

Supplementary Movie 2: The fluctuations and contour detection is shown for reticulocytes in presence of Pfs25 (negative control).

Supplementary Movie 3: The fluctuations and contour detection is shown for reticulocytes in presence of PVP01_0000100 CTD.
