# Supplementary material for: The structure of a Plasmodium vivax Tryptophan Rich Antigen domain suggests a lipid binding function for a pan-Plasmodium multi-gene family
Source: Nat Commun. 2023 Sep 14;14:5703. doi: 10.1038/s41467-023-40885-8 (PMC10502043; doi:10.1038/s41467-023-40885-8)
Supplement: Supplementary file 7 — Reporting Summary [file 41467_2023_40885_MOESM7_ESM.pdf]

nature portfolio

Corresponding author(s): Julian C Rayner, Janet E. Deane, Manoj Duraisingh

Last updated by author(s): Jul 23, 2023

Reporting Summary

Nature Portfolio wishes to improve the reproducibility of the work that we publish. This form provides structure for consistency and transparency in reporting. For further information on Nature Portfolio policies, see our Editorial Policies and the Editorial Policy Checklist.

Statistics

For all statistical analyses, confirm that the following items are present in the figure legend, table legend, main text, or Methods section.

n/a	Confirmed	
☐	☒	The exact sample size (*n*) for each experimental group/condition, given as a discrete number and unit of measurement
☐	☒	A statement on whether measurements were taken from distinct samples or whether the same sample was measured repeatedly
☐	☒	The statistical test(s) used AND whether they are one- or two-sided<br>*Only common tests should be described solely by name; describe more complex techniques in the Methods section.*
☒	☐	A description of all covariates tested
☐	☒	A description of any assumptions or corrections, such as tests of normality and adjustment for multiple comparisons
☐	☒	A full description of the statistical parameters including central tendency (e.g. means) or other basic estimates (e.g. regression coefficient) AND variation (e.g. standard deviation) or associated estimates of uncertainty (e.g. confidence intervals)
☐	☒	For null hypothesis testing, the test statistic (e.g. *F*, *t*, *r*) with confidence intervals, effect sizes, degrees of freedom and *P* value noted<br>*Give P values as exact values whenever suitable.*
☒	☐	For Bayesian analysis, information on the choice of priors and Markov chain Monte Carlo settings
☒	☐	For hierarchical and complex designs, identification of the appropriate level for tests and full reporting of outcomes
☐	☒	Estimates of effect sizes (e.g. Cohen's *d*, Pearson's *r*), indicating how they were calculated

Our web collection on statistics for biologists contains articles on many of the points above.

Software and code

Policy information about availability of computer code

Data collection	No customized algorithm or software has been used in this study
Data analysis	Microscopy Image analysis: Zen Blue and ImageJ<br>Flow Cytometry: FlowJo Ver10 and BD FACS Diva<br>Flickering Spectrometry for reticulocyte membrane tension measurement and wrapping energy calculation: Matlab, the scripts for the analysis are reported in Kariuki, Menendez and Introini et al., 2020, Nature (10.1038/s41586-020-2726-6)<br>Phylogenetic tree generation and analysis: PhyML<br>Structure solution: Phaser was used for molecular replacement using a model generated from RoseTTAFold. ArpWarp was used to build an initial model of PVP01_0000100_CTD, followed by manual building in Coot. Structure refinement was carried out with Coot, ISOLDE and phenix.refine.<br>Secondary Structure prediction: NetSurfP 3.0<br>Programme used for modeling: RoseTTAFold and Alfafold<br>All constructs, primers and GuideRNA design for gene editing using CRISPR: Benchling<br>Manuscript Figure Preparation: Adobe Illustrator<br>For plotting data, preparing graphs and statistical analysis: GraphPAD Prism 9.

For manuscripts utilizing custom algorithms or software that are central to the research but not yet described in published literature, software must be made available to editors and reviewers. We strongly encourage code deposition in a community repository (e.g. GitHub). See the Nature Portfolio guidelines for submitting code & software for further information.

Data

Policy information about availability of data

All manuscripts must include a data availability statement. This statement should provide the following information, where applicable:

- Accession codes, unique identifiers, or web links for publicly available datasets
- A description of any restrictions on data availability
- For clinical datasets or third party data, please ensure that the statement adheres to our policy

The authors declare that the data supporting the findings of this study are available within Main Text, Supplementary Figures, Tables and videos. Additional data generated during the peer review process are provided in the peer review file. The structure factors and atomic coordinates for PVP01_0000100 C terminal domain are deposited in the PDB under the accession code 8ARL (https://www.rcsb.org/structure/unreleased/8ARL). Source data are provided with this paper.

Research involving human participants, their data, or biological material

Policy information about studies with human participants or human data. See also policy information about sex, gender (identity/presentation), and sexual orientation and race, ethnicity and racism.

Reporting on sex and gender	Not Applicable to this study
Reporting on race, ethnicity, or other socially relevant groupings	Not Applicable to this study
Population characteristics	Not Applicable to this study
Recruitment	Not Applicable to this study
Ethics oversight	Not Applicable to this study

Note that full information on the approval of the study protocol must also be provided in the manuscript.

Field-specific reporting

Please select the one below that is the best fit for your research. If you are not sure, read the appropriate sections before making your selection.

[x] Life sciences [ ] Behavioural & social sciences [ ] Ecological, evolutionary & environmental sciences

For a reference copy of the document with all sections, see nature.com/documents/nr-reporting-summary-flat.pdf

Life sciences study design

All studies must disclose on these points even when the disclosure is negative.

Sample size	For red blood cell binding assays and Reticulocyte Invasion assays in flow cytometry 100,000 total events were counted. The number of cells recorded here is quite standard for invasion and binding assays in malaria field. There was no statistical methods used to predetermine the sample size.
Data exclusions	None
Replication	Reproducibility was confirmed by performing independent experiments with a minimum of 2-3 biological replicates with at least 2-3 technical replicates as per the nature of the experiment. The number of replicates, sample size and statistical information are mentioned in the respective sections of the manuscript.
Randomization	1. The binding and invasion study was performed using reticulocytes isolated from fresh blood (less than 48 hour) and repeated with different batches of blood from different individuals. Blood was ordered from a commercial supplier, with no specifications about age/gender/genetics etc, so were from random participants.<br><br>2. Each binding assay was performed with freshly purified protein samples, to avoid bias for a specific batch of proteins.
Blinding	1. The erythrocyte and reticulocyte samples were obtained from commercial suppliers. Researchers are blinded to the donors, who are anonymised, and researchers have no access to any information about them.<br>2. For the IFA experiment the preparation of cells and staining and imaging was done in an alternate way by two co-authors independently.<br>3. The lipid binding experiment is performed by two co-authors independently with two different assays (lipid dot blot and liposome binding assay) without knowing the outcome.<br>4. For the flickering spectroscopy assay the co-author was blinded about the protein samples.

Reporting for specific materials, systems and methods

We require information from authors about some types of materials, experimental systems and methods used in many studies. Here, indicate whether each material, system or method listed is relevant to your study. If you are not sure if a list item applies to your research, read the appropriate section before selecting a response.

Materials & experimental systems

n/a	Involved in the study
☐	☒ Antibodies
☐	☒ Eukaryotic cell lines
☒	☐ Palaeontology and archaeology
☒	☐ Animals and other organisms
☒	☐ Clinical data
☒	☐ Dual use research of concern
☒	☐ Plants

Methods

n/a	Involved in the study
☒	☐ ChIP-seq
☐	☒ Flow cytometry
☒	☐ MRI-based neuroimaging

Antibodies

Antibodies used	All the antibodies used in the study has been reported in the methods section of the manuscript. Primary antibodies were generated against Plasmodium vivax TRAgs in rabbit. The antibodies were generated from Eurogentech, Belgium as follows:<br>PVP01_0948700 (ZGB21026)<br>PVP01_0404200 (ZGB21048)<br>PVP01_0202200 (ZGB21025)<br>PVP01_0000100 (ZGB21027)<br>Plasmodium knowlesi merozoite surface marker anti-PkMSP1-19 raised in rat (kind donation from Ellen Knuepfer), AntiCD71-PE (cat. no. 12-0711-82, Thermo) , anti-His-rabbit HRP (Proteintech, Ptglab, USA). goat anti rabbit Alexa Fluor 633 (Thermo scientific, #A21070) and goat anti rat Alexa Fluor 488 (Thermo Scientific, #A11006).
Validation	ELISA studies have been been performed against each purified protein of interest to assess the antibody titer and specificity by the company Eurogentech. The specificity of the rabbit antibodies against its target were further assessed in the manuscript by IFA (Figure 1 a, b,c, Figure 5c and Supplementary Fig.3) and Western Blot (Figure 1, Supplementary figure 2). The primary rabbit antibody dilution used for western Blot is 1:5000. For IFA antibody dilutions were ranged from 1:200 to 1:2000 were tried and 1:750 was found to be the optimized dilution in which the images were taken.<br>Plasmodium knowlesi merozoite surface marker Rat anti-PkMSP1-19 (Ndegwa et al., 2021 Plos Pathogens, (https://doi.org/10.1371/journal.ppat.1008864))<br>Anti CD71-PE (Cho et al.,2015 Cell Death and Differentiation,(https://doi.org/10.1038/cdd.2014.230))<br>anti-His-rabbit HRP (Yang et al., 2018 Nature Communication (https://doi.org/10.1038/s41467-018-03588-z)).

Eukaryotic cell lines

Policy information about cell lines and Sex and Gender in Research

Cell line source(s)	Mammalian cell line used: HEK293E and HEK293F.
Authentication	No formal authentication for the cell lines were performed.
Mycoplasma contamination	Mycoplasma contamination was not verified.
Commonly misidentified lines (See ICLAC register)	No Commonly misidentified lines were used in the study.

Flow Cytometry

Plots

Confirm that:

☒ The axis labels state the marker and fluorochrome used (e.g. CD4-FITC).

☒ The axis scales are clearly visible. Include numbers along axes only for bottom left plot of group (a 'group' is an analysis of identical markers).

☐ All plots are contour plots with outliers or pseudocolor plots.

☒ A numerical value for number of cells or percentage (with statistics) is provided.

Methodology

Sample preparation	A detailed methodology of the red blood cell binding assay and Reticulocyte Invasion assay is described in the material method section of the manuscript.
Instrument	BD LSR Fortessa
Software	To analyse the flow results two different softwares were used: FlowJo Ver10 and BDFACS DIVA.
Cell population abundance	Flow cytometry experiments did not include sorting.
Gating strategy	For binding assays: Gate 1: The cells were selected based on forward (FSC-A on Y axis) and side scatter (SSC-A on X axis)<br>Gate 2: singlets were selected based on FSC-H(Y axis) and FSC-A (X axis)<br>Gate 3: Quadrant gate (Q1,Q2,Q3 and Q4) was set based on the red blood cell population only (Stained with Thiazole Orange (488_530/30A_Y axis) and cell Trace Far Red stain (561_582/15A_X axis)<br><br>For reticulocyte invasion assay: The quadrant gate was set based on the red blood cell population infected with parasites (SyBR green positive) followed by anti CD71 staining showing how many of the red blood cell population (parasite Infected vs non infected) are CD71 positive.<br>The gating populations and strategy were mentioned in the main text (Figure 2a) as well as in the supplementary file (Supplementary Figure 7 and 13)

☒ Tick this box to confirm that a figure exemplifying the gating strategy is provided in the Supplementary Information.
